# Association of low-frequency and rare coding variants with information processing speed

**DOI:** 10.1038/s41398-021-01736-6

**Published:** 2021-12-04

**Authors:** Jan Bressler, Gail Davies, Albert V. Smith, Yasaman Saba, Joshua C. Bis, Xueqiu Jian, Caroline Hayward, Lisa Yanek, Jennifer A. Smith, Saira S. Mirza, Ruiqi Wang, Hieab H. H. Adams, Diane Becker, Eric Boerwinkle, Archie Campbell, Simon R. Cox, Gudny Eiriksdottir, Chloe Fawns-Ritchie, Rebecca F. Gottesman, Megan L. Grove, Xiuqing Guo, Edith Hofer, Sharon L. R. Kardia, Maria J. Knol, Marisa Koini, Oscar L. Lopez, Riccardo E. Marioni, Paul Nyquist, Alison Pattie, Ozren Polasek, David J. Porteous, Igor Rudan, Claudia L. Satizabal, Helena Schmidt, Reinhold Schmidt, Stephen Sidney, Jeannette Simino, Blair H. Smith, Stephen T. Turner, Sven J. van der Lee, Erin B. Ware, Rachel A. Whitmer, Kristine Yaffe, Qiong Yang, Wei Zhao, Vilmundur Gudnason, Lenore J. Launer, Annette L. Fitzpatrick, Bruce M. Psaty, Myriam Fornage, M. Arfan Ikram, Cornelia M. van Duijn, Sudha Seshadri, Thomas H. Mosley, Ian J. Deary

**Affiliations:** 1 Human Genetics Center, School of Public Health, University of Texas Health Science Center at Houston, Houston, TX USA; 2 Lothian Birth Cohorts, University of Edinburgh, Edinburgh, UK; 3 Department of Psychology, University of Edinburgh, Edinburgh, UK; 4 Icelandic Heart Association, Kopavogur, Iceland; 5 Gottfried Schatz Research Center for Cell Signaling, Metabolism and Aging, Medical University of Graz, Graz, Austria; 6 Cardiovascular Health Research Unit, Department of Medicine, University of Washington, Seattle, WA USA; 7 Brown Foundation Institute of Molecular Medicine, University of Texas Health Science Center at Houston, Houston, TX USA; 8 Medical Research Council Human Genetics Unit, Institute of Genetics and Cancer, University of Edinburgh, Edinburgh, UK; 9 Generation Scotland, Centre for Genomic and Experimental Medicine, Institute of Genetics and Cancer, University of Edinburgh, Edinburgh, UK; 10 Department of General Internal Medicine, Johns Hopkins University School of Medicine, Baltimore, MD USA; 11 Department of Epidemiology, School of Public Health, University of Michigan, Ann Arbor, MI USA; 12 Survey Research Center, Institute for Social Research, University of Michigan, Ann Arbor, MI USA; 13 Department of Epidemiology, Erasmus MC University Medical Center, Rotterdam, The Netherlands; 14 Department of Neurology, Sunnybrook Health Sciences Centre, Toronto, ON Canada; 15 Department of Medicine, University of Toronto, Toronto, ON Canada; 16 Department of Biostatistics, Boston University School of Public Health, Boston, MA USA; 17 Framingham Heart Study, Framingham, MA USA; 18 Department of Neurology and Alzheimer Center, Amsterdam Neuroscience, Vrieje Universiteit Medical Center, Amsterdam, The Netherlands; 19 Human Genome Sequencing Center, Baylor College of Medicine, Houston, TX USA; 20 Centre for Genomic and Experimental Medicine, Institute of Genetics and Cancer, University of Edinburgh, Edinburgh, UK; 21 Usher Institute, University of Edinburgh, Edinburgh, UK; 22 Health Data Research UK, University of Edinburgh, Edinburgh, UK; 23 Department of Neurology, Johns Hopkins University School of Medicine, Baltimore, MD USA; 24 Department of Epidemiology, Johns Hopkins University Bloomberg School of Public Health, Baltimore, MD USA; 25 The Institute for Translational Genomics and Population Sciences, Department of Pediatrics, The Lundquist Institute for Biomedical Innovation at Harbor-UCLA Medical Center, Torrance, CA USA; 26 Clinical Division of Neurogeriatrics, Department of Neurology, Medical University of Graz, Graz, Austria; 27 Institute for Medical Informatics, Statistics and Documentation, Medical University of Graz, Graz, Austria; 28 Department of Neurology, University of Pittsburgh School of Medicine, Pittsburgh, PA USA; 29 Department of Psychiatry, University of Pittsburgh School of Medicine, Pittsburgh, PA USA; 30 Faculty of Medicine, University of Split, Split, Croatia; 31 Gen-Info Ltd, Zagreb, Croatia; 32 Centre for Cognitive Ageing and Cognitive Epidemiology, University of Edinburgh, Edinburgh, UK; 33 Centre for Global Health Research, Usher Institute for Population Health Sciences and Informatics, University of Edinburgh, Edinburgh, UK; 34 Glenn Biggs Institute for Alzheimer’s and Neurodegenerative Diseases, UT Health San Antonio, San Antonio, TX USA; 35 Department of Neurology, Boston University School of Medicine, Boston, MA USA; 36 Kaiser Permanente Northern California Division of Research, Oakland, CA USA; 37 Department of Data Science, University of Mississippi Medical Center, Jackson, MS USA; 38 Division of Population Health and Genomics, Ninewells Hospital and Medical School, University of Dundee, Dundee, UK; 39 Division of Nephrology and Hypertension, Mayo Clinic, Rochester, MN USA; 40 Department of Psychiatry, University of California San Francisco, San Francisco, CA USA; 41 Faculty of Medicine, University of Iceland, Reykjavik, Iceland; 42 Laboratory of Epidemiology and Population Science, National Institute on Aging, Intramural Research Program, National Institutes of Health, Bethesda, MD USA; 43 Department of Family Medicine, University of Washington, Seattle, WA USA; 44 Department of Epidemiology, University of Washington, Seattle, WA USA; 45 Department of Health Services, University of Washington, Seattle, WA USA; 46 Department of Public Health, Oxford University, Oxford, UK; 47 Division of Geriatrics, Department of Medicine, University of Mississippi Medical Center, Jackson, MS USA

**Keywords:** Medical genetics, Molecular neuroscience

## Abstract

Measures of information processing speed vary between individuals and decline with age. Studies of aging twins suggest heritability may be as high as 67%. The Illumina HumanExome Bead Chip genotyping array was used to examine the association of rare coding variants with performance on the Digit-Symbol Substitution Test (DSST) in community-dwelling adults participating in the Cohorts for Heart and Aging Research in Genomic Epidemiology (CHARGE) Consortium. DSST scores were available for 30,576 individuals of European ancestry from nine cohorts and for 5758 individuals of African ancestry from four cohorts who were older than 45 years and free of dementia and clinical stroke. Linear regression models adjusted for age and gender were used for analysis of single genetic variants, and the T5, T1, and T01 burden tests that aggregate the number of rare alleles by gene were also applied. Secondary analyses included further adjustment for education. Meta-analyses to combine cohort-specific results were carried out separately for each ancestry group. Variants in *RNF19A* reached the threshold for statistical significance (*p* = 2.01 × 10^−6^) using the T01 test in individuals of European descent. *RNF19A* belongs to the class of E3 ubiquitin ligases that confer substrate specificity when proteins are ubiquitinated and targeted for degradation through the 26S proteasome. Variants in *SLC22A7* and *OR51A7* were suggestively associated with DSST scores after adjustment for education for African-American participants and in the European cohorts, respectively. Further functional characterization of its substrates will be required to confirm the role of *RNF19A* in cognitive function.

## Introduction

Cognitive function can be classified into a number of domains such as reasoning, memory, verbal ability and information processing speed [1]. Measures of processing speed vary between individuals and decline on average with age [2, 3]. Low scores on the Digit-Symbol Substitution Test (DSST), the psychometric test examined in the current analysis, have been associated with both incident mild cognitive impairment and dementia [4, 5]. In addition to being considered as a possible endophenotype for age-related neurological disorders [6, 7] and other psychiatric conditions such as schizophrenia and attention deficit hyperactivity disorder [8, 9], processing speed has sometimes been seen as a relatively basic cognitive function that explains some of the differences in other cognitive abilities [10]. Since heritability evaluated in twin studies has been estimated to be as high as 67% for inter-individual variation in performance on tests of processing speed [11–14], three genome-wide association studies (GWAS) have been performed to identify common genetic variants that may contribute to this cognitive phenotype [15–17]. To date, there is evidence for genome-wide association with the rs17518584 variant located within an intron of *CADM2* and information processing speed in a sample of 32,070 older adults of European ancestry [17], whereas no significant associations were found in two smaller studies in which there were 4038 participants from four cohorts [15] or 1086 young adults [16]. More recently, associations of intronic single nucleotide polymorphisms (SNPs) in *SH2B3* (rs10849947) and *SPATS2* (rs10931898) and reaction time measured using a computerized game were detected in a GWAS that included 111,483 individuals in UK Biobank. Twenty three genes were also significantly associated with reaction time in the same study in gene-based analyses [18].

An exome genotyping array containing over 200,000 coding variants discovered through exome sequencing in ~12,000 individuals has become available to comprehensively evaluate rare coding variants. Variants that affect protein structure were selected if they were found in two or more individuals in more than two sequencing projects, and thus collectively, the array represents nearly all non-synonymous coding and splice variation with a > 1:1000 allele frequency in the European population [19]. The goal of this study was to test the hypothesis that rare coding variants in addition to common genetic polymorphisms contribute to scores on a test of processing speed in non-demented community-dwelling adults by combining results across studies participating in the Cohorts for Heart and Aging Research in Genomic Epidemiology (CHARGE) Consortium [20].

## Materials and methods

### Study populations

Nine population-based epidemiological cohort studies contributed to the discovery phase of the analysis: Age, Gene/Environment Susceptibility–Reykjavik Study (AGES-Reykjavik); Atherosclerosis Risk in Communities (ARIC) Study; Cardiovascular Health Study (CHS); Coronary Artery Risk Development in Young Adults (CARDIA); CROATIA-Korčula study (Korčula); Generation Scotland: Scottish Family Health Study (GS:SFHS); Genetic Epidemiology Network of Arteriopathy (GENOA); Lothian Birth Cohort 1921 (LBC1921); and Lothian Birth Cohort 1936 (LBC1936); Details for each cohort are described in the Supplementary Material. Written informed consent was obtained from all participants, and the investigators in each of the cohort studies obtained approval from their institutional review board or equivalent committee. All individuals in the study were 45 years or older and determined to be free of stroke and dementia using criteria established within each individual cohort. The total sample size was 36,334 and included 30,576 individuals of European ancestry and 5758 African-Americans. Replication was sought in 1697 individuals of European descent from two independent cohorts: Austrian Stroke Prevention Study (ASPS) and Rotterdam Study (RS).

### Genotyping

Most of the study participants were genotyped using the HumanExome Bead Chip v1.0 (Illumina, Inc. San Diego, CA), and variant calling was performed jointly for AGES, ARIC, CARDIA, CHS, GENOA, and RS at the University of Texas Health Science Center at Houston [19]. LBC1921, LBC1936, and CROATIA-Korčula were called in Genome Studio (Illumina Inc.) based on the CHARGE Consortium joint calling cluster file. Quality control procedures included checking concordance with previously collected GWAS genotyping data; exclusion of individuals missing more than 5% of genotypes; population clustering outliers; those with high inbreeding coefficients, rates of heterozygosity, or unexpectedly high identity-by-descent; and participants with gender mismatches. All genetic variants were coded additively with respect to the minor allele in the jointly called dataset. GS:SFHS was genotyped using the HumanExome Bead Chip v1_A and variant calling was performed using GenCall. ASPS genotyping was performed at the Helmholtz Zentrum München using the Illumina HumanExome v1.1 chip and Genome Studio Version V2011.1 software. Samples were excluded if there was contamination with other DNA, sex mismatch, cryptic relatedness, excess heterozygosity, duplicates on the chip or low call rate (<95%). SNPs were excluded based on low call rate (<95%) and Hardy–Weinberg equilibrium *p* value < 10^−6^.

### Cognitive tests

The DSST of the Wechsler Adult Intelligence Scale-Revised (WAIS-R) [21] requires the participant to translate numbers (1–9) to symbols using a key provided at the top of the test page. The WAIS-R was used by ARIC, CHS, and GENOA and was scored as the number of correct translations completed within 90 s. The duration of the version of the test [22] administered by AGES and CROATIA-Korčula was 90 s for AGES, and was 120 s for CROATIA-Korčula. GS, LBC1921, and LBC1936 used the test from the WAIS-III UK [23] with a test duration of 120 s. CARDIA administered the test from the WAIS III [24] with the score calculated as the number correct within 90 s. For all of the DSST tests, a higher score indicates a higher measure of cognition.

### Statistical analysis

Single variant and gene-based inverse variance meta-analyses were conducted using the seqMeta package (https://cran.r-project.org/web/packages/seqMeta/seqMeta.pdf). For the single variant analyses, non-synonymous and splice variants with a minor allele frequency (MAF) > 0.1% were evaluated for both African-American study participants and individuals of European ancestry. In the gene-based analyses using the T5, T1, and T01 tests that aggregate the total number of rare alleles at each locus [25], results were filtered using a cumulative MAF ≥ 0.05%, and were limited to genes in which there were at least 2 variants contributing to the test. These three tests incorporated non-synonymous and splice site variants with a MAF of <5%, <1%, and <0.01%, respectively. Two different statistical models were applied in all cohorts. In the first model, linear regression models were adjusted for age, gender, study center if appropriate, family relationship if appropriate, and principal components to correct for population structure with cognitive test scores examined as quantitative traits. The second model included all of the covariates specified in the first model and was further adjusted for educational attainment. Effective sample size (N-weighted) meta-analyses were carried out for individuals of European ancestry due to the different DSST protocols used among the various cohorts and were performed using METAL [26] after initially conducting two separate inverse variance meta-analyses for groups categorized on the basis of test duration (90 s and 120 s). For African-Americans, all cohorts included in the discovery set used a common test duration so inverse variance weighted meta-analysis was performed. A two-sided *p* value < 0.05/number of single variants (European ancestry: *N* = 51,043 variants, *p* < 9.8 × 10^−7^; African ancestry: *N* = 78,701 variants, *p* < 6.4 × 10^−7^) or <0.05/20,000 genes (*p* < 2.5 × 10^−6^) was considered statistically significant after Bonferroni correction for multiple comparisons.

### Candidate genes

The meta-analysis results for single variants were examined for association with low-frequency polymorphisms in genes previously associated with processing speed [17], or recently identified in studies of Alzheimer’s disease using either an exome-wide genotyping array or whole exome sequencing [27–33].

### Gene expression, gene-set enrichment, and molecular network analyses

Gene expression in human tissues was assessed using the Genotype Tissue expression portal (GTEx, Broad Institute of MIT and Harvard, Cambridge, MA; (http://www.gtexportal.org/home) [34]. Differential gene expression in multiple brain regions over the human lifespan was explored using data from the Human Brain Transcriptome Project (http://hbatlas.org/pages/hbtd) [35]. Summary statistics from the meta-analyses of the individuals of European ancestry and African ancestry were analyzed separately using FUnctional Mapping and Annotation of genetic associations (FUMA) to explore enrichment in biological pathways [36]. Evidence for overrepresentation of prioritized genes in gene sets represented in the MsigDB [37] and WikiPathways [38] databases was obtained using the hypergeometric test implemented in the GENE2FUNC function. Genes reaching a *p* value ≤ 1 × 10^−4^ in either the single variant or gene-based meta-analyses adjusted for age and gender (model 1), or age, gender, and education (model 2) were included in the analyses. The same genes were also considered the focus or input genes in the gene network analyses conducted using the core analysis function in Ingenuity Pathway Analysis (IPA) software (QIAGEN Inc., https://www.qiagenbioinformatics.com/products/ingenuity-pathway-analysis) to generate a set of networks based on the relationships between these and other molecules cataloged in the Ingenuity Knowledge Base [39]. The resulting networks are scored using a right tailed Fisher’s exact test and the −log_10_ (*p* value) to test the null hypothesis that the association of the focus genes and a set of genes selected from the database and added to the network is due to chance. A score > 1.3 (*p* < 0.05) was chosen as the a priori level of statistical significance. The IPA core analysis function was also used to identify biological functions associated with the focus genes.

## Results

The demographic characteristics, mean DSST score, and the DSST test duration for each cohort contributing results to either the discovery or replication meta-analyses are shown in Table 1 stratified by ancestry. Educational attainment was classified into 4–5 categories to include the lowest and highest levels of education.as appropriate in each study (Supplementary Table 1). Quantile-quantile plots revealed no inflation of test statistics for any of the individual cohorts or for the meta-analyses (Supplementary Figs. 1, 2).

**Table 1:** Discovery and replication cohorts.

Cohort	N^a^	N^b^	Age (years) (mean, SD)	Female (%)	DSST Test duration (seconds)	DSST (digit-symbol pairs) (mean, SD)
*Discovery European ancestry*						
AGES	2583	2583	75.90 ± 5.49	57.99	90	29.85 ± 14.10
ARIC	10 201	10 201	57.21 ± 5.68	53.39	90	49.09 ± 11.46
CARDIA	1694	1692	50.73 ± 3.37	53.36	90	74.68 ± 14.73
CHS	3754	3754	72.53 ± 5.42	57.03	90	38.89 ± 12.52
GENOA	940	940	59.94 ± 9.48	59.15	90	50.92 ± 12.65
GS	9559	8744	51.86 ± 13.45	59.23	120	70.41 ± 17.02
Korčula	665	629	55.37 ± 10.08	62.86	120	50.75 ± 20.26
LBC1921	245	245	83.35 ± 0.54	58.59	120	42.75 ± 12.98
LBC1936	935	935	69.54 ± 0.84	49.42	120	56.94 ± 12.83
Total	30 576	29 723				
*Discovery African ancestry*						
ARIC	3103	3103	56.11 ± 5.72	64.45	90	31.33 ± 13.23
CARDIA	1319	1315	49.49 ± 3.84	59.67	90	64.34 ± 15.72
CHS	639	639	72.00 ± 5.03	62.91	90	30.10 ± 12.55
GENOA	697	697	61.82 ± 9.38	72.17	90	33.65 ± 14.28
Total	5758	5754				
*Replication European ancestry*					LDST Test duration (seconds)	
RS	1561	1546	72.64 ± 6.86	48.94	60	26.33 ± 7.11
ASPS	136	136	70.92 ± 6.86	62.50	60	25.40 ± 6.62
Total	1697	1682				

*SD* standard deviation, *DSST* Digit-Symbol Substitution Test, *LDST* Letter Digit Substitution Test.

N^a^ number of participants contributing to meta-analysis adjusted for age and gender.

N^b^ number of participants contributing to meta-analysis adjusted for age, gender, and educational attainment.

When low-frequency variants were tested individually for association with performance on the DSST, no polymorphic loci that met the a priori significance thresholds were identified for either ancestry group under an additive genetic model.

In the gene-based analyses adjusting for age and gender, there was one genome-wide significant result (*p* < 2.5 × 10^−6^) for the ring finger protein 19A, RBR E3 protein ubiquitin ligase (*RNF19A*) gene on chromosome 8q22 that was associated with DSST scores (*p* = 2.01 × 10^−6^) in individuals of European ancestry using the T01 test (Table 2, Supplementary Table 2), although this association was attenuated after further adjustment for education (*p* = 6.31 × 10^−6^) (Table 2, Supplementary Table 3). *RNF19A* has previously been implicated in Parkinson’s disease in which there is slowed information processing [40, 41] and in amyotrophic lateral sclerosis [42, 43]. Examination of the GTEx tissue expression data (Supplementary Fig. 3) revealed that *RNF19A* was most highly expressed in the endocervix, testis, uterus and bladder, whereas it appeared to be transcribed at a relatively low level in all brain regions analyzed. When *RNF19A* was evaluated in the human brain across the lifespan (Supplementary Fig. 4), there was evidence of differential expression in some brain regions. Expression of *RNF19A* was highest in the first year of life in the neocortex and medial nucleus of the thalamus, whereas in the cerebellar cortex the level of *RNF19A* was lowest during the same time period and reached its maximum in early adulthood.

**Table 2:** Gene-based analysis of association of low-frequency variants and DSST scores.

Ancestry	Test	Gene	Chr	CMAF (%)	Beta^a^ (SE)	Beta^b^ (SE)	Variants (*N*)	Z-Score	*p* value^1^	Z-Score	*p* value^2^
*Genome-Wide*											
EA	T01	*RNF19A*	8q22.2	0.18	–	–	–	−4.75	2.01 × 10^−6^	−4.51	6.31 × 10^−6^
*Suggestive*											
EA	T5/T1	*OR51A7*	11p15.4	0.25	–	–	–	−4.42	9.76 × 10^−6^	−4.66	3.13 × 10^−6^
*Suggestive*											
AA	T01	*SLC22A7*	6p21.1	0.078	17.74 (3.97)	15.79 (3.37)	2	–	8.12 × 10^−6^	–	2.78 × 10^−6^

*DSST* Digit-Symbol Substitution Test, *EA* European ancestry, *AA* African ancestry, *Chr* chromosome, *CMAF* cumulative minor allele frequency of variants identified in burden test, *SE* standard error, *N* number of variants contributing to burden test, *Z-score* summary value for sample size weighted meta-analysis.

^1^*p* value adjusted for age and gender; ^2^*p* value adjusted for age, gender, and educational attainment.

^a^Beta beta coefficient for model adjusted for age and gender.

^b^Beta beta coefficient for model adjusted for age, gender, and educational attainment.

In addition, olfactory receptor family 51 subfamily A member 7 (*OR51A7*; chromosome 11p15.4) was suggestively associated with scores on the DSST using both the T5 test (*p* = 3.13 × 10^−6^) and T1 test (*p* = 3.13 × 10^−6^) after adjustment for education in the European cohort participants (Table 2, Supplementary Table 3). Two variants in solute carrier family 22 member 7 (*SLC22A7*) contributed to a suggestive association with performance on the DSST in African-American individuals using the T01 test only when educational attainment was added as a covariate to the regression models (*β* = 15.79 (SE = 3.37) number–symbol pairs; *p* = 2.78 × 10^−6^) (Table 2, Supplementary Tables 4, 5). The protein encoded by *SLC22A7* is involved in facilitative transport of cGMP and other guanine nucleotides in multiple tissues [44].

Replication was attempted for genes meeting the threshold for genome-wide significance in the gene-based tests after meta-analyzing the results for 1561 participants in the RS and 136 participants in the ASPS that used the Letter Digit Substitution Test (LDST) [45, 46], a related test of processing speed previously shown to be correlated with the DSST (*r* = 0.87, *p* < 0.01) in a group of 102 volunteers [17]. There was no evidence of association with performance on the LDST in these cohorts (Table 3).

**Table 3:** Replication analysis of association of genes and DSST scores—RS and ASPS (*N* = 1682).

Gene	Test	Beta (SE)	*p*^a^	CMAF	Variants (*N*)
*RNF19A*	T01	−44 (1.92)	0.82	0.0033	4

*DSST* Digit-Symbol Substitution Test, *Beta* beta coefficient, *SE* standard error, *p* p value, *CMAF* cumulative minor allele frequency of variants identified in burden test, *N* number of variants contributing to burden test.

^a^Adjusted for age and gender.

In study participants of both ethnicities, the meta-analysis results were examined for rare single nucleotide variants in the gene for cell adhesion molecule 2 (*CADM2*), a locus previously identified in a large GWAS of processing speed [17], and in several candidate genes previously reported to be associated with Alzheimer’s disease [27–33]. Among the variants that were included on the exome array, there was one nominally significant association with A-kinase anchoring protein 9 (*AKAP9*) rs149979685 (*β* = −3.117 (SE = 1.538) number–symbol pairs; *p* = 0.043) in African-Americans after adjusting for age and gender that did not survive adjustment for multiple comparisons (Supplementary Tables 6, 7). The *AKAP9* genetic variant was initially identified by whole exome sequencing in an African-American discovery cohort of Alzheimer’s disease cases and controls [27]. In addition, SNPs with a *p* value ≤ 10^−4^ in the meta-analyses of the results for the discovery cohorts in the earlier CHARGE consortium GWAS of processing speed [17] and that were also present on the exome array were evaluated for association with performance on the DSST in participants of both ancestries (Supplementary Table 8). Two exonic variants in ankyrin repeat and kinase domain containing 1 (*ANKK1*) met these criteria in both the meta-analysis adjusted for age and gender and the meta-analysis adjusted for age, gender, and educational attainment, but were not significantly associated with DSST scores in the current study using the exome array genotyping data (all *p* ≥ 2.98 × 10^−3^).

Gene-set enrichment analysis of genes prioritized using FUMA, and IPA network analysis were also performed separately by ancestry to assess shared biological functions. Enrichment in immune system-related pathways was found for both African-Americans and individuals of European ancestry, consistent with the discovery of a central role for the immune response in genetic studies of the risk of Alzheimer’s disease [31, 47]. In the cohorts of European ancestry, *RNF19A* was one of five genes that overlapped with the tested gene set for the Reactome adaptive immune pathway [48]. Enrichment in a KEGG calcium-signaling pathway [49] was seen only in African-Americans (Supplementary Table 9). IPA network analysis in African-Americans revealed that the most highly scored network (*p* = 1 × 10^−23^) was associated with cardiovascular disease development and function, organismal development, and tissue morphology and included an indirect interaction between *SLC22A7* and the nonessential amino acid L-glutamic acid. In individuals of European ancestry, the most highly scored network (*p* = 1 × 10^−25^) included *RNF19A* and was associated with embryonic development, organismal development, and tissue development. Supplementary Figs. 5, 6 show the network diagrams generated separately by IPA for each ancestry group. In addition, Supplementary Table 10 shows the most significantly associated biological functions related to the focus genes used to produce the molecular networks and that were detected in the IPA core analysis for each ancestry group. The top biological function in the physiological systems development category in African-Americans was nervous system development with 5 associated genes (*p* value range = 4.77 × 10^−2^–8.51 × 10^−4^) and neurological disease was the second most highly associated biological function in the diseases and disorders category for individuals of European descent with 14 associated genes (*p* value range = 2.82 × 10^−2^–4.80 × 10^−5^).

## Discussion

When the exome array was used to evaluate 30,576 individuals of European descent and 5758 African-Americans, only *RNF19A* was found to exceed the a priori threshold for genome-wide significant association with DSST scores in European ancestry cohorts after conducting the T01 test and adjusting for age and gender. Ubiquitination of proteins targeted for degradation through the 26S proteasome requires the successive activity of an E1 ubiquitination-activation enzyme, an E2 ubiquitin-conjugating enzyme, and an E3 ubiquitin ligase [50, 51]. Mutations in E3 ubiquitin ligases have previously been reported to be associated with both common and rare neurological disorders including autism spectrum disorder and Angelman syndrome [52]. *RNF19A* is a RING finger-type E3 ubiquitin ligase [53] that has been shown to localize to Lewy bodies, a characteristic neuronal inclusion in the brain of patients with Parkinson’s disease, and to ubiquitylate synphylin-1. Synphilin-1 was demonstrated to interact in a yeast two-hybrid screen with α-synuclein, another component of Lewy bodies known to cause neuronal degeneration when overexpressed in transgenic flies and mice [42]. *RNF19A* also appears to play a role in familial amyotrophic lateral sclerosis (ALS) by ubiquitylating mutant superoxide dismutase (SOD-1) proteins and promoting their degradation, thereby contributing to the protection of surviving motor neurons [43]. In addition, *Rnf19a*-deficient mice have been found to have reduced adult neurogenesis and enhanced long-term potentiation in the dentate gyrus [54]. There were no genome-wide significant results identified for African-Americans using any of the gene-based tests.

Though it is possible to speculate that efficient quality control of cellular proteins mediated by *RNF19A* is implicated in processing speed in cognitively normal individuals, the identity of its substrate targets and the stage of development during which it may influence cognitive function are currently unknown. Whereas many of the previous reports described above indicate that *RNF19A* is expressed in neurons in humans, their primary focus was the role of *RNF19A* in neuronal inclusions containing insoluble protein aggregates that are not found in the absence of a neurodegenerative disease [42, 43, 53, 55–58]. The results of the network analysis suggest that *RNF19A* may play a role in embryonic development, and interacts with several genes that have been identified in GWAS of either Alzheimer’s disease or vascular risk factors associated with cognitive decline in late life. A direct interaction between *RNF19A* and nuclear receptor coactivator 3 (*NCOA3*) was observed. *NCOA3*, a member of the p160 steroid coactivator (SRC) family that modulates transcriptional activation by nuclear receptors in response to hormones, has been implicated in retinoic acid signaling in mouse fetal cortical neurons and is expressed in the murine and human adult brain [59–63]. An intronic variant in *NCOA3* (rs13042367) was recently reported to be associated with HDL-cholesterol levels in a GWAS of circulating lipoproteins [64, 65]. Variants in other interaction partners of *NCOA3* including insulin growth factor 1 (*IGF1* rs5742643), HNF1 homeobox A (*HNF1A* rs1800574 and rs56348580), and IQ motif containing K (*IQCK* rs7185636) were associated with systolic blood pressure [66, 67], type 2 diabetes [68–72], or Alzheimer’s disease [47], respectively. Links to genes implicated in Alzheimer’s disease were also found in the results of the gene expression network analysis for *CADM2* identified in the previous CHARGE GWAS of processing speed [17].

The strengths of the study include the well-phenotyped study populations, the representation of individuals of both European and African ancestry, and joint calling of the variants present on the exome array across the participating cohorts. In addition, this is to our knowledge the largest sample size reported for an analysis of rare genetic variants and a single test of processing speed. There are also limitations. The detection of a single gene associated with performance on the DSST suggests that an even larger study may be required to identify additional genome-wide significant findings as has been previously observed for common variants in GWAS of other complex traits, such as height and body mass index [73, 74]. Because the coding and splice site variants present on the exome array are only a subset of the total number of variants in the human genome, and since rare variants found in only one individual were not included by design, it is possible that analysis of whole exome or whole genome sequencing data will be required to fully characterize the role of low-frequency genetic variation in information processing speed.

## Supplementary information

Supplementary information

Supplementary table 1

Supplementary table 2

Supplementary table 3

Supplementary table 4

Supplementary table 5

Supplementary table 6

Supplementary table 7

Supplementary table 8

Supplementary table 9

Supplementary table 10

## Data Availability

Summary statistics for the meta-analyses will be available via dbGaP study accession phs000930.v9.p1 (CHARGE (Consortium for Heart and Aging Research in Genomic Epidemiology) Consortium Summary Results from Genomic Studies).
