# Correction: Association of low-frequency and rare coding variants with information processing speed

**DOI:** 10.1038/s41398-022-01852-x

**Published:** 2022-03-01

**Authors:** Jan Bressler, Gail Davies, Albert V. Smith, Yasaman Saba, Joshua C. Bis, Xueqiu Jian, Caroline Hayward, Lisa Yanek, Jennifer A. Smith, Saira S. Mirza, Ruiqi Wang, Hieab H. H. Adams, Diane Becker, Eric Boerwinkle, Archie Campbell, Simon R. Cox, Gudny Eiriksdottir, Chloe Fawns-Ritchie, Rebecca F. Gottesman, Megan L. Grove, Xiuqing Guo, Edith Hofer, Sharon L. R. Kardia, Maria J. Knol, Marisa Koini, Oscar L. Lopez, Riccardo E. Marioni, Paul Nyquist, Alison Pattie, Ozren Polasek, David J. Porteous, Igor Rudan, Claudia L. Satizabal, Helena Schmidt, Reinhold Schmidt, Stephen Sidney, Jeannette Simino, Blair H. Smith, Stephen T. Turner, Sven J. van der Lee, Erin B. Ware, Rachel A. Whitmer, Kristine Yaffe, Qiong Yang, Wei Zhao, Vilmundur Gudnason, Lenore J. Launer, Annette L. Fitzpatrick, Bruce M. Psaty, Myriam Fornage, M. Arfan Ikram, Cornelia M. van Duijn, Sudha Seshadri, Thomas H. Mosley, Ian J. Deary

**Affiliations:** 1 Human Genetics Center, School of Public Health, University of Texas Health Science Center at Houston, Houston, TX USA; 2 Lothian Birth Cohorts, University of Edinburgh, Edinburgh, UK; 3 Department of Psychology, University of Edinburgh, Edinburgh, UK; 4 Icelandic Heart Association, Kopavogur, Iceland; 5 Gottfried Schatz Research Center for Cell Signaling, Metabolism and Aging, Medical University of Graz, Graz, Austria; 6 Cardiovascular Health Research Unit, Department of Medicine, University of Washington, Seattle, WA USA; 7 Brown Foundation Institute of Molecular Medicine, University of Texas Health Science Center at Houston, Houston, TX USA; 8 Medical Research Council Human Genetics Unit, Institute of Genetics and Cancer, University of Edinburgh, Edinburgh, UK; 9 Generation Scotland, Centre for Genomic and Experimental Medicine, Institute of Genetics and Cancer, University of Edinburgh, Edinburgh, UK; 10 Department of General Internal Medicine, Johns Hopkins University School of Medicine, Baltimore, MD USA; 11 Department of Epidemiology, School of Public Health, University of Michigan, Ann Arbor, MI USA; 12 Survey Research Center, Institute for Social Research, University of Michigan, Ann Arbor, MI USA; 13 Department of Epidemiology, Erasmus MC University Medical Center, Rotterdam, The Netherlands; 14 Department of Neurology, Sunnybrook Health Sciences Centre, Toronto, ON Canada; 15 Department of Medicine, University of Toronto, Toronto, ON Canada; 16 Department of Biostatistics, Boston University School of Public Health, Boston, MA USA; 17 Framingham Heart Study, Framingham, MA USA; 18 Department of Neurology and Alzheimer Center, Amsterdam Neuroscience, Vrieje Universiteit Medical Center, Amsterdam, The Netherlands; 19 Human Genome Sequencing Center, Baylor College of Medicine, Houston, TX USA; 20 Centre for Genomic and Experimental Medicine, Institute of Genetics and Cancer, University of Edinburgh, Edinburgh, UK; 21 Usher Institute, University of Edinburgh, Edinburgh, UK; 22 Health Data Research UK, University of Edinburgh, Edinburgh, UK; 23 Department of Neurology, Johns Hopkins University School of Medicine, Baltimore, MD USA; 24 Department of Epidemiology, Johns Hopkins University Bloomberg School of Public Health, Baltimore, MD USA; 25 The Institute for Translational Genomics and Population Sciences, Department of Pediatrics, The Lundquist Institute for Biomedical Innovation at Harbor-UCLA Medical Center, Torrance, CA USA; 26 Clinical Division of Neurogeriatrics, Department of Neurology, Medical University of Graz, Graz, Austria; 27 Institute for Medical Informatics, Statistics and Documentation, Medical University of Graz, Graz, Austria; 28 Department of Neurology, University of Pittsburgh School of Medicine, Pittsburgh, PA USA; 29 Department of Psychiatry, University of Pittsburgh School of Medicine, Pittsburgh, PA USA; 30 Faculty of Medicine, University of Split, Split, Croatia; 31 Gen-Info Ltd, Zagreb, Croatia; 32 Centre for Cognitive Ageing and Cognitive Epidemiology, University of Edinburgh, Edinburgh, UK; 33 Centre for Global Health Research, Usher Institute for Population Health Sciences and Informatics, University of Edinburgh, Edinburgh, UK; 34 Glenn Biggs Institute for Alzheimer’s and Neurodegenerative Diseases, UT Health San Antonio, San Antonio, TX USA; 35 Department of Neurology, Boston University School of Medicine, Boston, MA USA; 36 Kaiser Permanente Northern California Division of Research, Oakland, CA USA; 37 Department of Data Science, University of Mississippi Medical Center, Jackson, MS USA; 38 Division of Population Health and Genomics, Ninewells Hospital and Medical School, University of Dundee, Dundee, UK; 39 Division of Nephrology and Hypertension, Mayo Clinic, Rochester, MN USA; 40 Department of Psychiatry, University of California San Francisco, San Francisco, CA USA; 41 Faculty of Medicine, University of Iceland, Reykjavik, Iceland; 42 Laboratory of Epidemiology and Population Science, National Institute on Aging, Intramural Research Program, National Institutes of Health, Bethesda, MD USA; 43 Department of Family Medicine, University of Washington, Seattle, WA USA; 44 Department of Epidemiology, University of Washington, Seattle, WA USA; 45 Department of Health Services, University of Washington, Seattle, WA USA; 46 Department of Public Health, Oxford University, Oxford, UK; 47 Division of Geriatrics, Department of Medicine, University of Mississippi Medical Center, Jackson, MS USA

**Keywords:** Medical genetics, Molecular neuroscience

Correction to: *Translational Psychiatry* 10.1038/s41398-021-01736-6, published online 4 December 2021

The original version of this article unfortunately contained mistakes in the author affiliations. The original article has been corrected.

The correct affiliations are as follows:

Eric Boerwinkle^1,19^, Archie Campbell^20,21,22^, Rebecca F. Gottesman^23,24^, Megan L. Grove^1^, Xiuqing Guo^25^, Edith Hofer^26,27^, Maria J. Knol^13^, Marisa Koini^26^, Oscar L. Lopez^28,29^, Riccardo E. Marioni^20^, Paul Nyquist^23^, Ozren Polasek^30,31^, David J. Porteous^20,32^, Igor Rudan^33^, Claudia L. Satizibal^17,34,35^, Reinhold Schmidt^26^, Stephen Sidney^36^, Jeannette Simino^37^, Blair H. Smith^38^, Stephen T. Turner^39^, Sven J. van der Lee^13^, Erin B. Ware^12^, Rachel A. Whitmer^36^, Kristine Yaffe^40^, Vilmundur Gudnason^4,41^, Lenore J. Launer^42^, Annette L. Fitzpatrick^43,44^, Bruce M. Psaty^6,44,45^, M. Arfan Ikram^13^, Cornelia M. van Duijn^13,46^, Sudha Seshadri^17,34,35^, Thomas H. Mosley^47^.

