# Supplementary material for: Association of low-frequency and rare coding variants with information processing speed
Source: Transl Psychiatry. 2021 Dec 4;11:613. doi: 10.1038/s41398-021-01736-6 (PMC8643353; doi:10.1038/s41398-021-01736-6)
Supplement: Supplementary file 1 — Supplementary information [file 41398_2021_1736_MOESM1_ESM.docx]

**SUPPLEMENTARY INFORMATION**

**Table of Contents**

Supplementary Notes

1. Cohorts

2. Supplementary Tables: The results for rare and low frequency genetic variants associated with performance on the Digit Substitution Test (DSST) are available as Supplementary Information, and the Supplementary Tables are listed below.

Supplementary Table 1. Educational attainment in participating cohorts

Supplementary Table 2. Meta-analysis of results for European ancestry cohorts (adjusted for age and gender; p < 1 x 10^-4^)

Supplementary Table 3. Meta-analysis of results for European ancestry cohorts (adjusted for age, gender, and education; p < 1 x 10^-4^)

Supplementary Table 4. Meta-analysis of results for African ancestry cohorts (adjusted for age and gender; p < 1 x 10^-4^)

Supplementary Table 5. Meta-analysis of results for African ancestry cohorts (adjusted for age, gender, and education; p < 1 x 10^-4^)

Supplementary Table 6. Association of single variants in *CADM2* and DSST scores

Supplementary Table 7. Association of single variants in Alzheimer’s disease candidate genes and DSST scores

Supplementary Table 8. Comparison of GWAS of DSST scores and analysis of rare variants using exome array

Supplementary Table 9. Gene-set enrichment analysis implemented using FUMA

Supplementary Table 10. Most significant diseases and biological functions identified in IPA core analysis

3. Supplementary Figures

Supplementary Figure 1: Quantile-Quantile (Q-Q) plots for discovery cohorts

Supplementary Figure 2: Quantile-Quantile (Q-Q) plots for discovery meta-analyses

Supplementary Figure 3: RNF19A expression data from GTEX. Data and figure accessed from the Genotype Tissue expression portal (GTex, Broad Institute of MIT and Harvard, Cambridge, MA; (http://www.gtexportal.org/home)

Supplementary Figure 4: Differential expression of RNF19A in human brain across lifespan. Data and figure accessed from the Human Brain Transcriptome project; (http://hbatlas.org/pages/hbtd)

Supplementary Figure 5: Network diagram generated by Ingenuity Pathway Analysis (European ancestry)

Supplementary Figure 6: Network diagram generated by Ingenuity Pathway Analysis (African-American)

4. Acknowledgements

**1. Cohorts**

**A. Discovery**

**Age, Gene/Environment Susceptibility-Reykjavik Study (AGES-Reykjavik):** The AGES-Reykjavik Study is a single center prospective cohort study based on the Reykjavik Study. The Reykjavik Study was initiated in 1967 by the Icelandic Heart Association to study cardiovascular disease and risk factors. The cohort included men and women born between 1907 and 1935 who lived in Reykjavik at the 1967 baseline examination. Re-examination of surviving members of the cohort was initiated in 2002 as part of the AGES-Reykjavik Study. The AGES-Reykjavik Study is designed to investigate aging using a multifaceted comprehensive approach that includes detailed measures of brain function and structure. All cohort members were European Caucasians. Briefly, as part of a comprehensive examination, all participants answered a questionnaire, underwent a clinical examination and had blood drawn.[^1^](#_ENREF_1) All consenting participants were offered a neuropsychological test battery[^2^](#_ENREF_2) including the DSST.[^3^](#_ENREF_3) The AGES-Reykjavik Study was approved by the National Bioethics Committee in Iceland (approval number, VSN-00-063) and by the National Institute on Aging intramural institutional review board. Written informed consent was obtained from all participants.

**Atherosclerosis Risk in Communities (ARIC) Study:** The ARIC study is a prospective population-based study of atherosclerosis and clinical atherosclerotic diseases in 15,792 men and women, including 11,478 white participants and 4,314 African American participants, drawn from 4 United States communities (Suburban Minneapolis, Minnesota; Washington County, Maryland; Forsyth County, North Carolina; and Jackson, Mississippi). In the first 3 communities, the sample reflects the demographic composition of the community. In Jackson, only black residents were enrolled. Participants were between age 45 and 64 years at their baseline examination in 1987-1989 when blood was drawn for DNA extraction and participants consented to genetic testing.[^4^](#_ENREF_4) Vascular risk factors and outcomes, including transient ischemic attack and stroke, were determined in a standard fashion.[^5^](#_ENREF_5) The second clinical examination of the ARIC Study cohort in 1990–1992 included a neuropsychological evaluation and tests administered included the DSST.[^6^](#_ENREF_6) [^7^](#_ENREF_7) Given the relatively young age of the cohort at the second examination (age range = 48-67 years) and low expected prevalence of dementia, a formal assessment for dementia was not performed. Written informed consent was provided by all study participants, and the study design and methods were approved by institutional review boards at each of the collaborating medical institutions: University of Mississippi Medical Center Institutional Review Board (Jackson Field Center); Wake Forest University Health Sciences Institutional Review Board (Forsyth County Field Center); University of Minnesota Institutional Review Board (Minnesota Field Center); and Johns Hopkins University School of Public Health Institutional Review Board (Washington County Field Center).

**Cardiovascular Health Study (CHS):** The CHS is a population-based cohort study of risk factors for vascular disease in adults 65 years or older conducted across 4 field centers in the United States: Sacramento County, California; Washington County, Maryland; Forsyth County, North Carolina; and Pittsburgh, Allegheny County, Pennsylvania.[^8^](#_ENREF_8) The original predominantly white cohort of 5,201 persons was recruited in 1989-1990 from a random sample of people on Medicare eligibility lists. An additional 687 African-Americans were enrolled in 1992-1993, for a total sample of 5,888. Vascular risk factors and outcomes, including transient ischemic attack, stroke and dementia, were determined in a standard fashion.[^9^](#_ENREF_9)^,^[^10^](#_ENREF_10) DNA was extracted from blood samples drawn on all participants who consented to genetic testing at their baseline examination in 1989-1990 or 1992-1993. As part of the CHS Cognition Study,[^10^](#_ENREF_10)^,^[^11^](#_ENREF_11) in 1992-1994 and again, in 1997-1999, participants were invited to undergo a detailed neuropsychological assessment including the DSST.[^7^](#_ENREF_7) The institutional review board at each university associated with the field centers (University of California, Davis; Johns Hopkins University; Wake Forest University; University of Pittsburgh) approved the study, and each participant gave informed consent.

**Coronary Artery Risk Development in Young Adults (CARDIA)**: The CARDIA study is a prospective multi-center investigation of the etiology and natural history of cardiovascular disease initiated in 1985-1986. The study’s initial enrollment consisted of 5,115 European American and African American men and women between 18 and 30 years old (52% African American and 55% women) recruited from 4 field centers (Birmingham, AL; Chicago, IL; Minneapolis, MN; and Oakland, CA). Detailed information about the CARDIA study design and methods of data collection have been previously published.[^12^](#_ENREF_12) At the year 25 follow-up examination, three standardized tests were administered to assess cognitive function including psychomotor speed using the DSST.[^13^](#_ENREF_13)^,^[^14^](#_ENREF_14) Written informed consent was provided by all study participants. The institutional review boards at each of the collaborating institutions (University of Alabama at Birmingham, Kaiser Permanente Northern California Division of Research, Northwestern University, University of Minnesota, and University of Texas Health Science Center at Houston) approved the study.

**CROATIA- Korcula study (Korcula):** The CROATIA-Korcula study[^15^](#_ENREF_15) includes 969 Croatians between the ages of 18 and 98. The field work was performed in 2007 and 2008 in the eastern part of the island, targeting healthy volunteers from the town of Korcula and the villages of Lumbarda, Zrnovo and Racisce. Ethical approval was obtained from appropriate regulatory bodies in both Scotland and Croatia and participants gave informed consent prior to joining the study. Study participants were invited to undergo a neuropsychological examination including the DSST.[^3^](#_ENREF_3)

**Generation Scotland: Scottish Family Health Study (GS:SFHS):** Generation Scotland: Scottish Family Health Study is a family-structured, population-based cohort study recruited between 2006 and 2011. All components of GS received ethical approval from the NHS Tayside Committee on Medical Research Ethics (REC Reference Number: 05/S1401/89). Regional sampling occurred in Glasgow, Tayside, Ayrshire, Arran, and North-East Scotland, yielding a total sample size of 24084 with an age range between 18 and 100 years and up to four generations per family. A full description of the cohort is provided elsewhere.[^16^](#_ENREF_16)^,^[^17^](#_ENREF_17) Four cognitive domains were assessed including processing speed using the DSST.[^18^](#_ENREF_18)

**Genetic Epidemiology Network of Arteriopathy (GENOA):** GENOA is a study of hypertensive sibships designed to investigate the genetic underpinnings of hypertension and target organ damage.[^19^](#_ENREF_19) In the initial phase of the GENOA study (Phase I: 1996-2001), all members of sibships containing ≥ 2 individuals with essential hypertension clinically diagnosed before age 60 were invited to participate, including both hypertensive and normotensive siblings (1,583 non-Hispanic whites from Rochester, MN, and 1,841 African Americans from Jackson, MS). The diagnosis of essential hypertension was established based on blood pressure levels measured at the study visit (>140 mmHg average systolic BP or >90 mmHg average diastolic BP) or a prior diagnosis of hypertension and current treatment with antihypertensive medications. Exclusion criteria were secondary hypertension, alcoholism or drug abuse, pregnancy, insulin-dependent diabetes mellitus, or active malignancy. In the second phase of the GENOA study (Phase II: 2000-2004), 1,241 white and 1,482 African American participants were successfully re-recruited to measure potential target organ damage due to hypertension. The Genetics of Microangiopathic Brain Injury (GMBI) study (2001-2006) is an ancillary study of GENOA undertaken to investigate susceptibility genes for ischemic brain injury. Phase II GENOA participants that had a sibling willing and eligible to participate in the GMBI study underwent a neurocognitive testing battery to assess several domains of cognitive function including the DSST.[^7^](#_ENREF_7) The study was approved by the Institutional Review Boards of Mayo Clinic, Rochester, MN, the University of Mississippi, Jackson, MS, and the University of Michigan, Ann Arbor, MI. Written informed consent was obtained from each participant.

**Lothian Birth Cohorts 1921 (LBC1921) and 1936 (LBC1936):** These studies include surviving participants from the Scottish Mental Survey of 1932 or 1947 (SMS1932 and SMS1947) who were born in 1921 (LBC1921) or 1936 (LBC1936), respectively.[^20-22^](#_ENREF_20) They were all Caucasian and almost all lived independently in the Lothian region (Edinburgh city and the surrounding area) of Scotland. The LBC1921 cohort comprised 550 members while the LBC1936 cohort included 1,091 participants. At mean age 83, LBC1921 participants underwent a neuropsychological examination including the Digit Symbol coding subtest from the Wechsler Adult Intelligence Scale-III UK.[^18^](#_ENREF_18) At age 70, LBC 1936 participants took a battery of cognitive tests including the DSST. Ethical approval for the study protocols was obtained from the Multi-Centre Research Ethics Committee for Scotland (MREC/01/0/56) and from the Lothian Research Ethics Committee (LREC/2003/7/23; LREC/2003/2/29). All subjects gave written, informed consent.

**B. Replication**

**Austrian Stroke Prevention Study (ASPS):** The Austrian Stroke Prevention Study (ASPS) is a single center prospective follow-up study on the effects of vascular risk factors on brain structure and function in the normal elderly population of the city of Graz, Austria. The procedure of recruitment and diagnostic work-up of study participants has been described previously.[^23^](#_ENREF_23)^,^[^24^](#_ENREF_24) A total of 2007 participants were randomly selected from the official community register stratified by gender and 5-year age groups. Individuals were excluded from the study if they had a history of neuropsychiatric disease, including previous stroke, transient ischemic attack, and dementia, or an abnormal neurologic examination determined on the basis of a structured clinical interview and a physical and neurologic examination. During two study periods between September 1991 and March 1994 and between January 1999 and December 2003 an extended diagnostic work-up including neuropsychological testing was done in 1,076 individuals aged 45 to 85 years randomly selected from the entire cohort: 509 from the first period and 567 from the second. In 1992 blood was drawn from all study participants. The neuropsychological test battery[^25^](#_ENREF_25) included among other tests the Letter Digit Substitution Task (LDST).[^26^](#_ENREF_26)^,^[^27^](#_ENREF_27) Approval was obtained from the local standard ethics committee of the Medical University of Graz. Written informed consent was obtained from all study participants.

**Rotterdam Study (RS):** The Rotterdam Study (RS) is a population-based cohort study among inhabitants of a district of Rotterdam (Ommoord), The Netherlands, and aims to examine the determinants of disease and health in the elderly with a focus on neurogeriatric, cardiovascular, bone, and eye disease.[^28^](#_ENREF_28)^,^[^29^](#_ENREF_29) In 1990-1993, 7,983 persons participated and were re-examined every 3 to 4 years (RS1). Additional cohorts from the same district were recruited in 2000-2001 (RS2, 3,011 subjects aged 55 years and older) and 2006-2008 (RS3, 3,932 subjects aged 45 years and older), with the exact same study protocol as RS1. All participants had DNA extracted at their first visit. Participants are constantly monitored for major events, including dementia and stroke, by automated linkage of the general practitioners’ records and hospital discharge files with the study database.[^30^](#_ENREF_30)^,^[^31^](#_ENREF_31) Participants in RS1 underwent several neuropsychological tests at follow-up examinations,[^32^](#_ENREF_32) including the LDST.[^26^](#_ENREF_26)^,^[^27^](#_ENREF_27) These tests were first assessed in RS1 in the third round of examinations (1997-1999) and were also administered in RS2 and RS3. The Rotterdam Study has been approved by the Medical Ethics Committee of the Erasmus MC (registration number MEC 02.1015) and by the Dutch Ministry of Health, Welfare and Sport (Population Screening Act WBO, license number 1071272-159521-PG). The Rotterdam Study Personal Registration Data collection is filed with the Erasmus MC Data Protection Officer under registration number EMC1712001. The Rotterdam Study has been entered into the Netherlands Trial Register (NTR; www.trialregister.nl) and into the WHO International Clinical Trials Registry Platform (ICTRP; https://apps.who.int/trialsearch/) under shared catalogue number NTR6831. All participants provided written informed consent to participate in the study and to have their information obtained from treating physicians.

References:

1. Harris TB, Launer LJ, Eiriksdottir G, Kjartansson O, Jonsson PV, Sigurdsson G, *et al.* Age, Gene/Environment Susceptibility-Reykjavik Study: multidisciplinary applied phenomics. *Am J Epidemiol* **165**, 1076-87 (2007).

2. Palm WM, Saczynski JS, van der Grond J, Sigurdsson S, Kjartansson O, Jonsson PV, *et al.* Ventricular dilation: association with gait and cognition. *Ann Neurol* **66**, 485-93 (2009).

3. Wechsler D. *Manual for the Wechsler Adult Intelligence Scale.*, (Psychological Corporation, New York, 1955).

4. The ARIC investigators. The Atherosclerosis Risk in Communities (ARIC) Study: design and objectives. *Am J Epidemiol* **129**, 687-702 (1989).

5. Rosamond WD, Folsom AR, Chambless LE, Wang CH, McGovern PG, Howard G, *et al.* Stroke incidence and survival among middle-aged adults: 9-year follow-up of the Atherosclerosis Risk in Communities (ARIC) cohort. *Stroke* **30**, 736-43 (1999).

6. Cerhan JR, Folsom AR, Mortimer JA, Shahar E, Knopman DS, McGovern PG, *et al.* Correlates of cognitive function in middle-aged adults. Atherosclerosis Risk in Communities (ARIC) Study Investigators. *Gerontology* **44**, 95-105 (1998).

7. Wechsler D. *Wechsler adult intelligence scale-revised*, (Psychological Corporation, New York, 1981).

8. Fried LP, Borhani NO, Enright P, Furberg CD, Gardin JM, Kronmal RA, *et al.* The Cardiovascular Health Study: design and rationale. *Ann Epidemiol* **1**, 263-76 (1991).

9. Longstreth WT Jr, Bernick C, Fitzpatrick A, Cushman M, Knepper L, Lima J, *et al.* Frequency and predictors of stroke death in 5,888 participants in the Cardiovascular Health Study. *Neurology* **56**, 368-75 (2001).

10. Lopez OL, Kuller LH, Fitzpatrick A, Ives D, Becker JT, Beauchamp N*.* Evaluation of dementia in the cardiovascular health cognition study. *Neuroepidemiology* **22**, 1-12 (2003).

11. Lopez OL, Becker JT, Jagust WJ, Fitzpatrick A, Carlson MC, DeKosky ST, *et al.* Neuropsychological characteristics of mild cognitive impairment subgroups. *J Neurol Neurosurg Psychiatry* **77**, 159-65 (2006).

12. Friedman GD, Cutter GR, Donahue RP, Hughes GH, Hulley SB, Jacobs DR Jr, *et al.* CARDIA: study design, recruitment, and some characteristics of the examined subjects. *J Clin Epidemiol* **41**, 1105-16 (1988).

13. Reis JP, Loria CM, Launer LJ, Sidney S, Liu K, Jacobs DR Jr, *et al.* Cardiovascular health through young adulthood and cognitive functioning in midlife. *Ann Neurol* **73**, 170-9 (2013).

14. Wechsler D. *Wechsler Adult Intelligence Scale-III (WAIS-III)*, (Psychological Corporation, New York, 1997).

15. Zemunik T, Boban M, Lauc G, Janković S, Rotim K, Vatavuk Z, *et al.* Genome-wide association study of biochemical traits in Korcula Island, Croatia. *Croat Med J* **50**, 23-33 (2009).

16. Smith BH, Campbell H, Blackwood D, Connell J, Connor M, Deary IJ, *et al.* Generation Scotland: the Scottish Family Health Study; a new resource for researching genes and heritability. *BMC Med Genet* **7**, 74 (2006).

17. Smith BH, Campbell A, Linksted P, Fitzpatrick B, Jackson C, Kerr SM, *et al.* Cohort Profile: Generation Scotland: Scottish Family Health Study (GS:SFHS). The study, its participants and their potential for genetic research on health and illness. *Int J Epidemiol* **42**, 689-700 (2013).

18. Wechsler D. *WAIS-III UK administration and scoring manual*, (Psychological Corporation, London, 1998).

19. FBPP Investigators. Multi-center genetic study of hypertension: The Family Blood Pressure Program (FBPP). *Hypertension* **39**, 3-9 (2002).

20. Deary IJ, Gow AJ, Taylor MD, Corley J, Brett C, Wilson V, *et al.* The Lothian Birth Cohort 1936: a study to examine influences on cognitive ageing from age 11 to age 70 and beyond. *BMC Geriatr* **7**, 28 (2007).

21. Deary IJ, Whiteman MC, Starr JM, Whalley LJ, Fox HC. The impact of childhood intelligence on later life: following up the Scottish mental surveys of 1932 and 1947. *J Pers Soc Psychol* **86**, 130-47 (2004).

22. Deary IJ, Gow AJ, Pattie A, Starr JM. Cohort profile: the Lothian Birth Cohorts of 1921 and 1936. *Int J Epidemiol* **41**, 1576-84 (2012).

23. Schmidt R, Fazekas F, Kapeller P, Schmidt H, Hartung HP. MRI white matter hyperintensities: three-year follow-up of the Austrian Stroke Prevention Study. *Neurology* **53**, 132-9 (1999).

24. Schmidt R, Lechner H, Fazekas F, Niederkorn K, Reinhart B, Grieshofer P, *et al.* Assessment of cerebrovascular risk profiles in healthy persons: definition of research goals and the Austrian Stroke Prevention Study (ASPS). *Neuroepidemiology* **13**, 308-13 (1994).

25. Schmidt R, Ropele S, Enzinger C, Petrovic K, Smith S, Schmidt H, *et al.* White matter lesion progression, brain atrophy, and cognitive decline: the Austrian stroke prevention study. *Ann Neurol* **58**, 610-6 (2005).

26. Jolles J, Houx P, van Boxtel M, Ponds R. *The Maastricht Aging Study: Determinants of cognitive aging*, (Neuropsych Publishers, Maastricht, 1995).

27. Lezak M. *Neuropsychological Assessment*, (Oxford University Press, New York, 1995).

28. Hofman A, Grobbee DE, de Jong PT, van den Ouweland FA. Determinants of disease and disability in the elderly: the Rotterdam Elderly Study. *Eur J Epidemiol* **7**, 403-22 (1991).

29. Ikram MA, Brusselle GGO, Murad SD, van Duijn CM, Franco OH, Goedegebure A, *et al.* The Rotterdam Study: 2018 update on objectives, design and main results. *Eur J Epidemiol* **32**, 807-850 (2017).

30. Bots ML*,* Looman SJ, Koudstaal PJ, Hofman A, Hoes AW, Grobbee DE. Prevalence of stroke in the general population. The Rotterdam Study. *Stroke* **27**, 1499-501 (1996).

31. Hollander M, Koudstaal PJ, Bots ML, Grobbee DE, Hofman A, Breteler MM. Incidence, risk, and case fatality of first ever stroke in the elderly population. The Rotterdam Study. *J Neurol Neurosurg Psychiatry* **74**, 317-21 (2003).

32. Prins ND, van Dijk EJ, den Heijer T, Vermeer SE, Jolles J, Koudstaal PJ, *et al.* Cerebral small-vessel disease and decline in information processing speed, executive function and memory. *Brain* **128**, 2034-41 (2005).

**3. Supplementary Figures**

**Supplementary Figure 1: Quantile-Quantile (Q-Q) plots for discovery cohorts**

**A.** European Ancestry, adjusted for age and gender


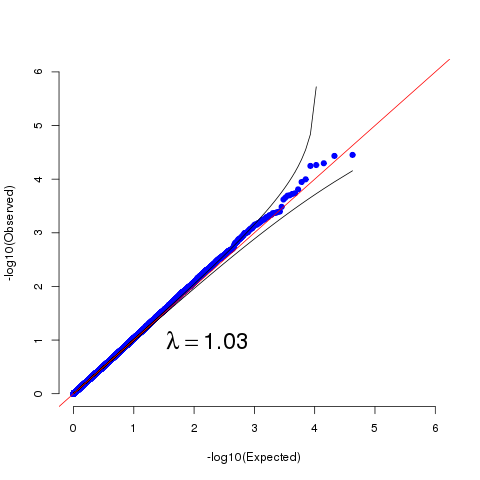

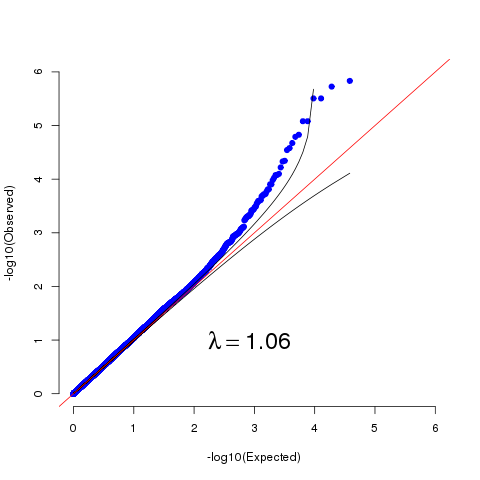

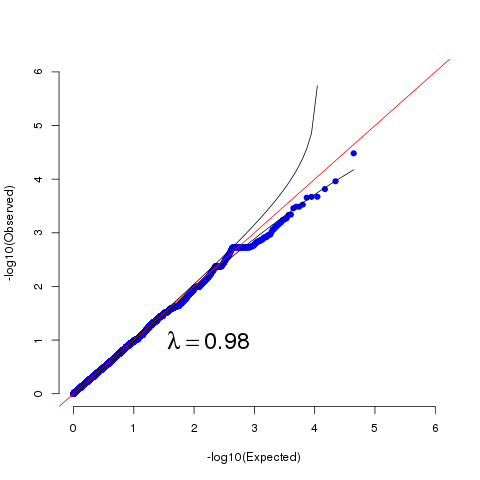

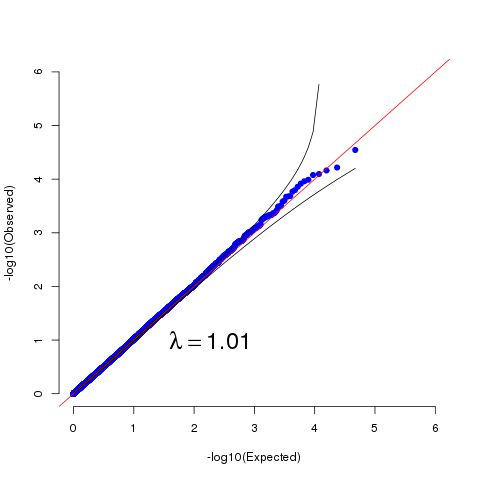

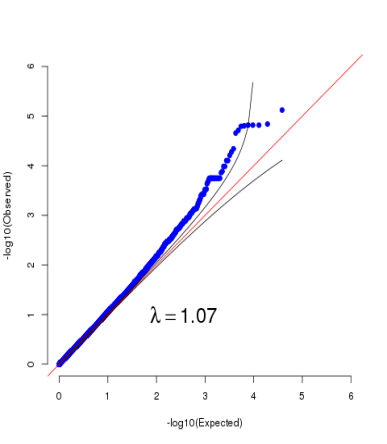

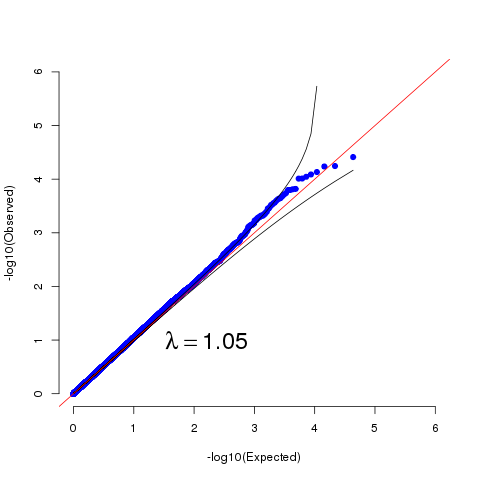

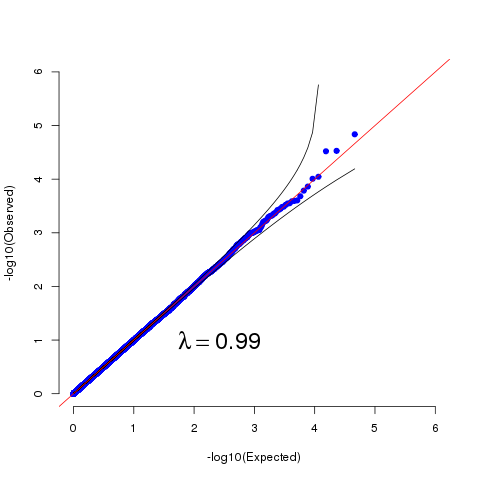

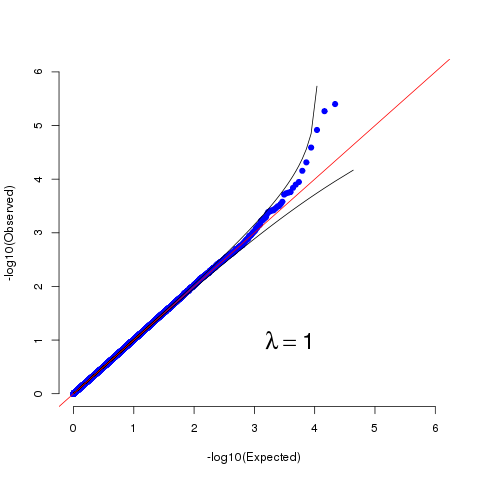

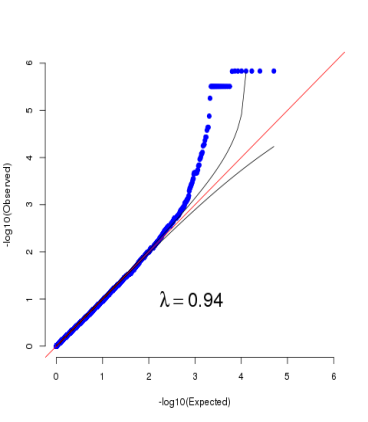


AGES ARIC CARDIA CHS GENOA

GS KORCULA LBC1921 LBC1936

**B.** European Ancestry, adjusted for age, gender, and education

GS KORCULA LBC1921 LBC1936


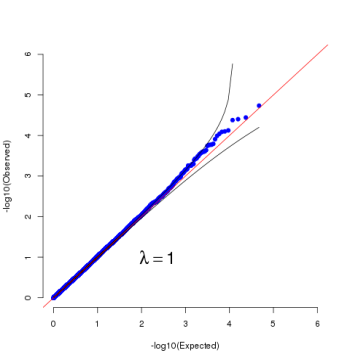

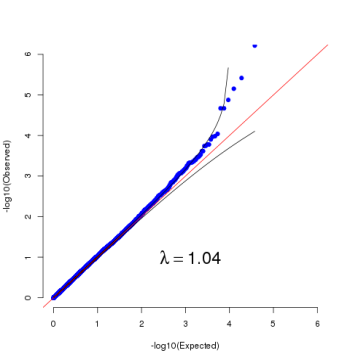

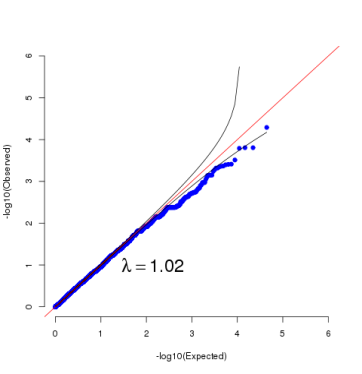

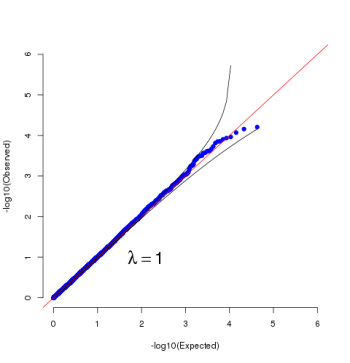

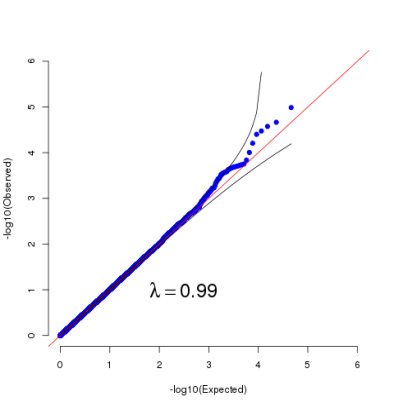

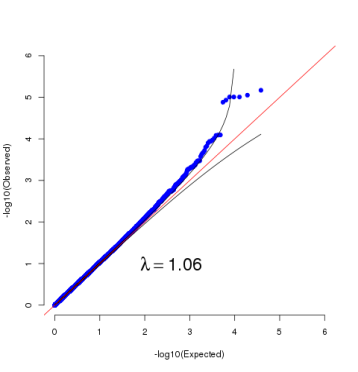

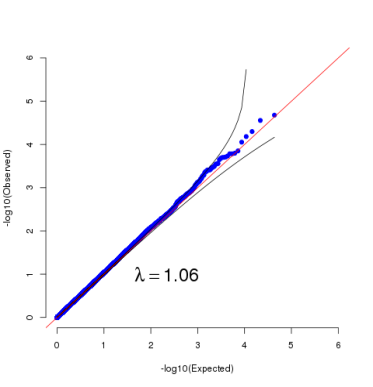

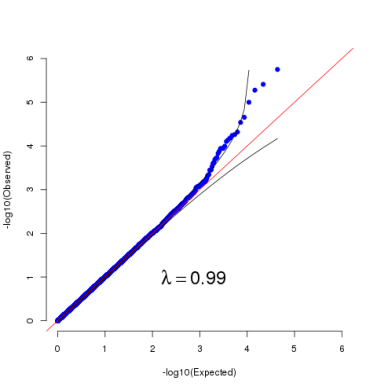

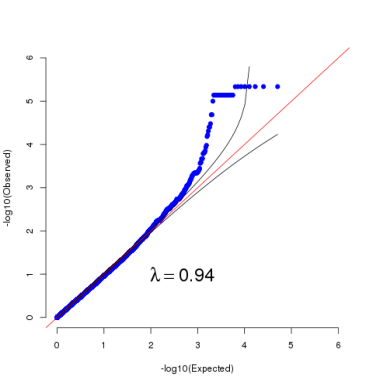


AGES ARIC CARDIA CHS GENOA

**C.** African-American, adjusted for age and gender


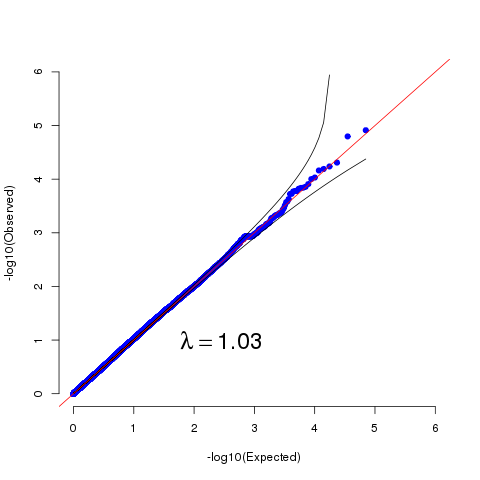

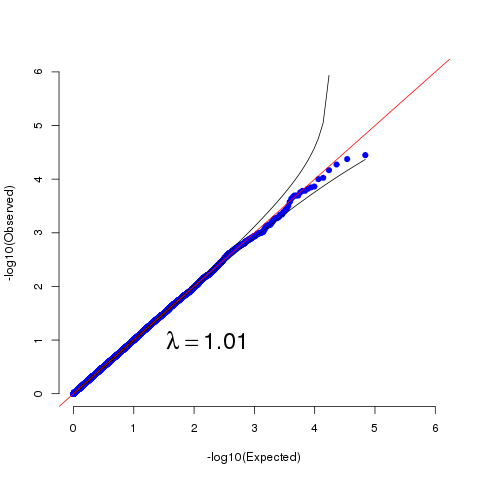

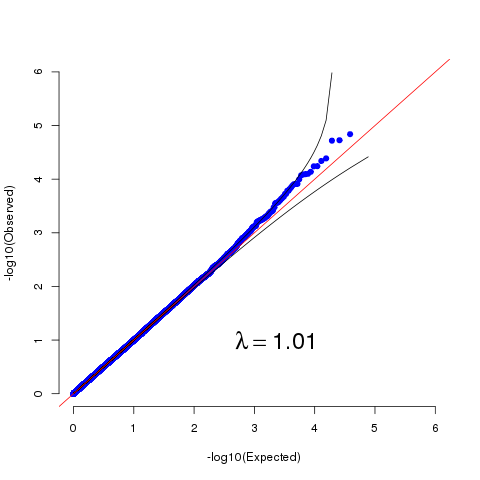

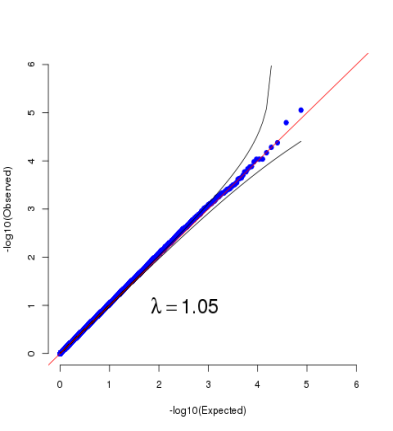


ARIC CARDIA CHS GENOA

**D.** African-American, adjusted for age, gender, and education


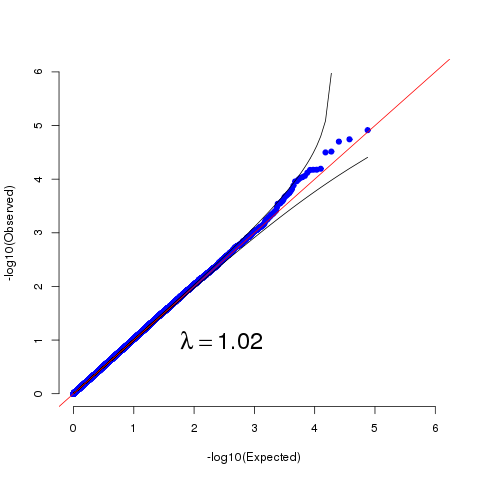

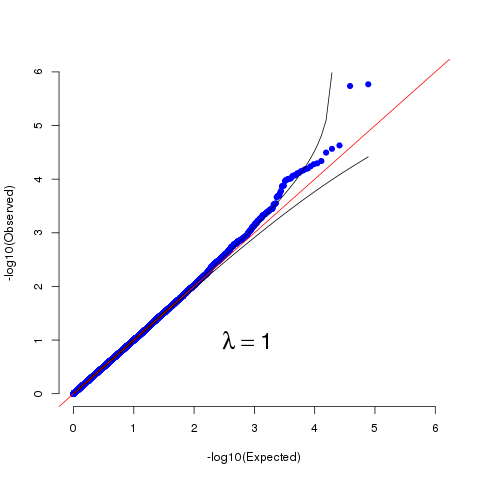

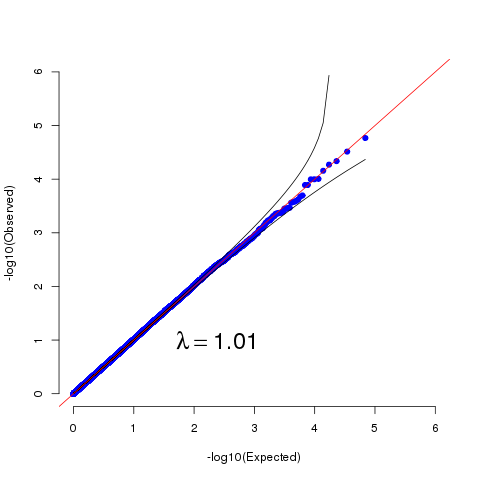

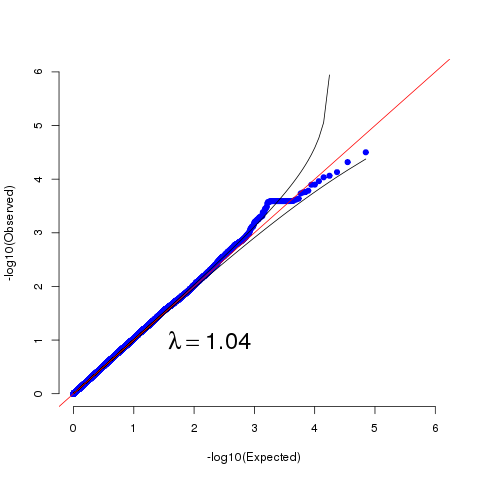


ARIC CARDIA CHS GENOA

**Supplementary Figure 2: Quantile-Quantile (Q-Q) plots for discovery meta-analyses**

**A.** European Ancestry, Manhattan Plot and Q-Q Plot, adjusted for age and gender, SNP


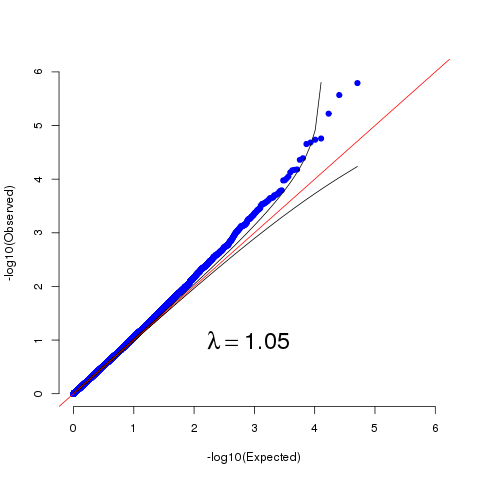

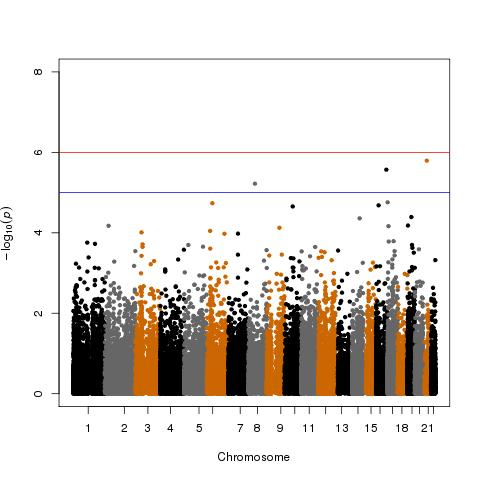


**B.** European Ancestry, Q-Q Plot, adjusted for age and gender, burden tests

T5 T1 T01


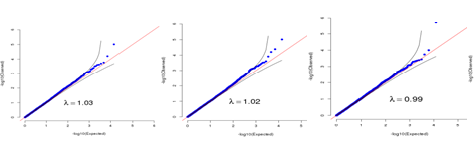


**C.** European Ancestry, Manhattan Plot and Q-Q Plot, adjusted for age, gender, education, SNP


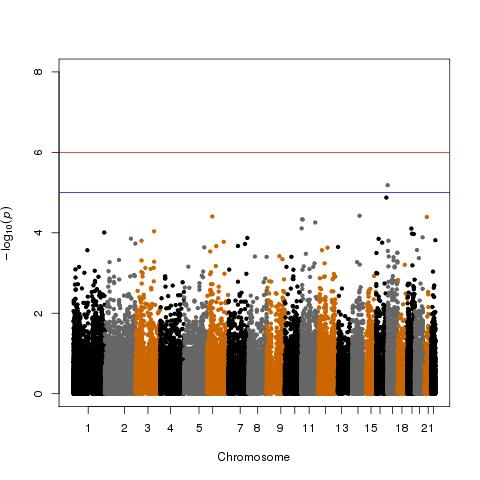

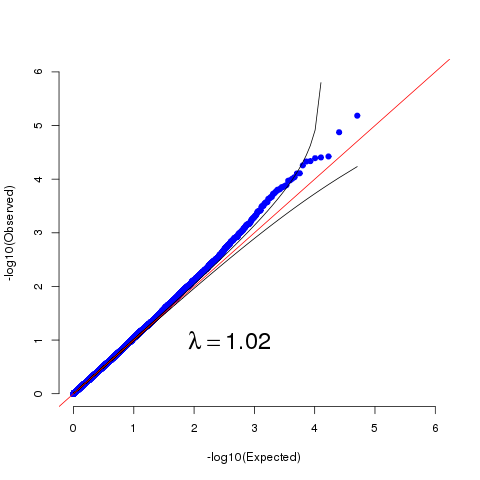


**D.** European Ancestry, Q-Q Plot, adjusted for age, gender, and education, burden tests

T5 T1 T01

**
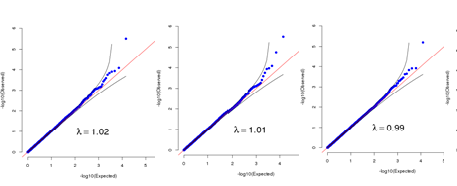
**

**E.** African American, Manhattan Plot and Q-Q Plot, adjusted for age and gender, SNP


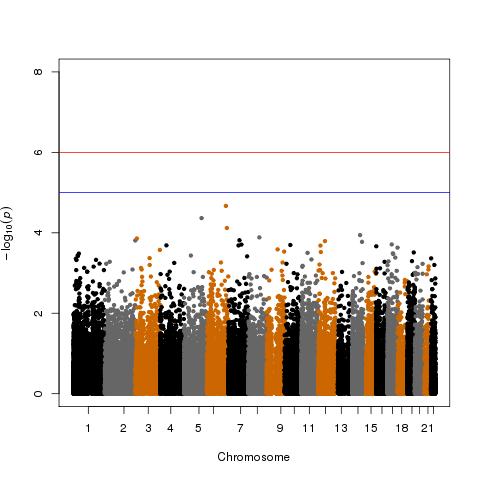

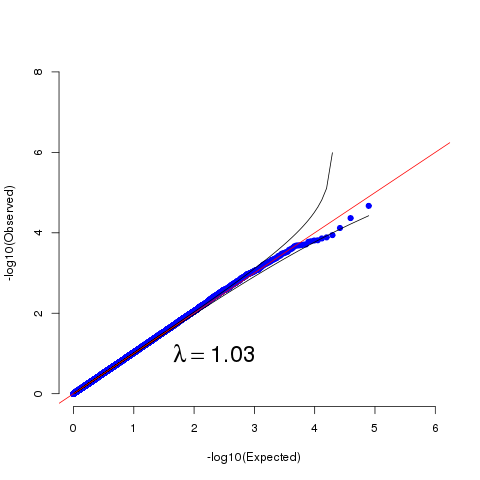


**F.** African American, Q-Q Plot, adjusted for age and gender, burden tests

T5 T1 T01

**
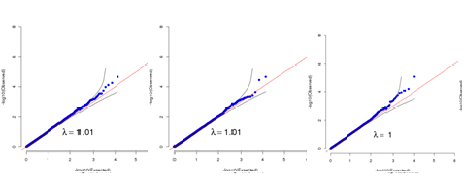
**

**G.** African-American, Manhattan Plot and Q-Q Plot, adjusted for age, gender, education, SNP


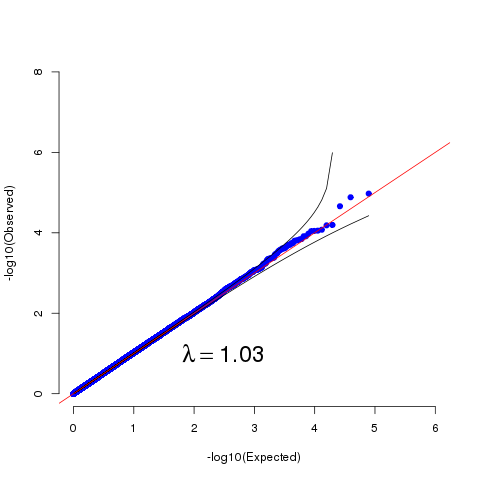

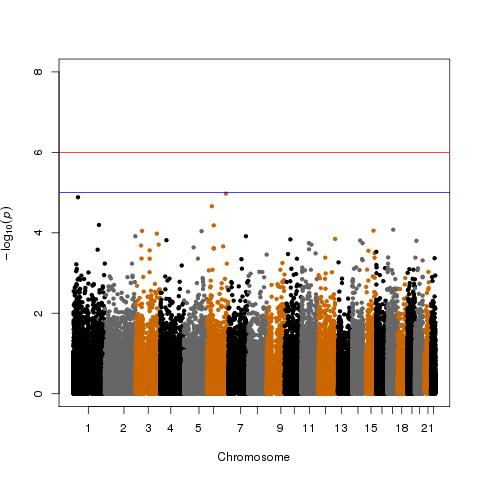


**H.** African-American, Q-Q Plot, adjusted for age, gender, and education, burden tests


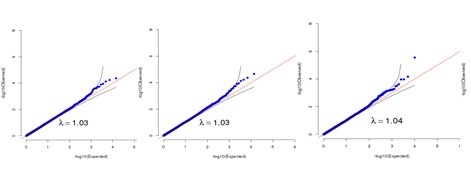


T5 T1 T01

**Supplementary Figure 3: *RNF19A* (Gencode ID ENSG00000034677.7) expression data from GTEx. Data and figure accessed from the Genotype Tissue expression portal (GTEx, Broad Institute of MIT and Harvard, Cambridge, MA; (http://www.gtexportal.org/home). The tissue examined is shown on the x-axis. Expression values are shown on the y-axis in TPM (Transcripts Per Million). Data source: GTEx Analysis Release V7**


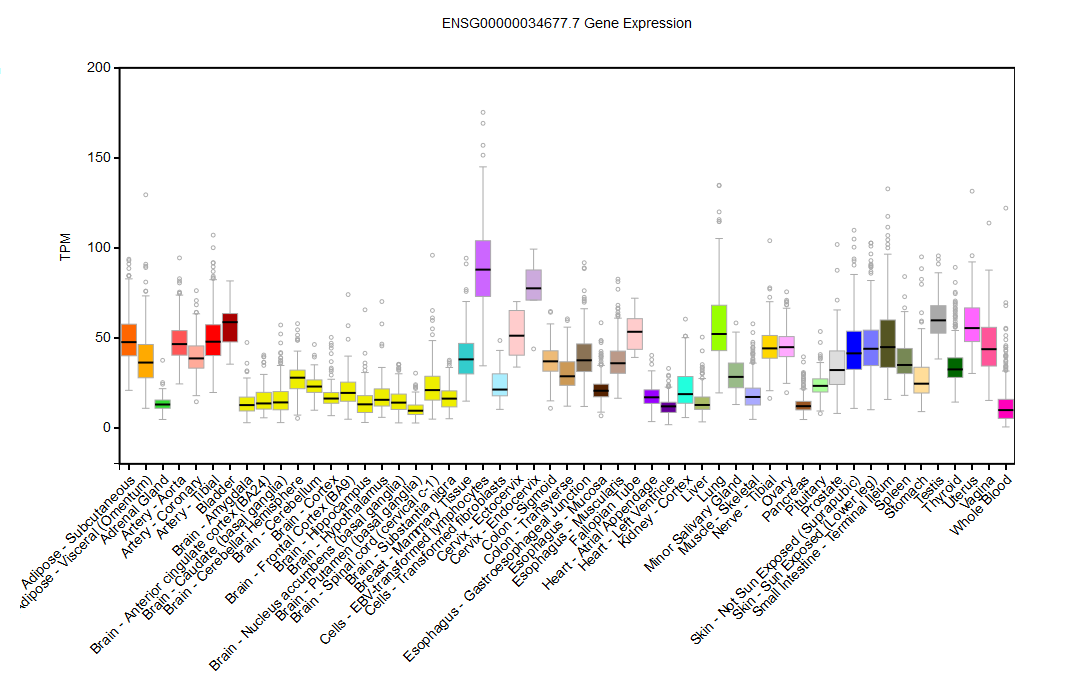


**Supplementary Figure 4: Differential expression of *RNF19A* in human brain across lifespan. Data and figure accessed from the Human Brain Transcriptome project (http://hbatlas.org/pages/hbtd) (**NCX, neocortex; HIP, hippocampus; AMY, amygdala; STR, striatum; MD, mediodorsal nucleus of thalamus; CBC, cerebellar cortex). Developmental age in days is displayed on the x-axis; mRNA signal intensity (log2) is shown on the y-axis.

**
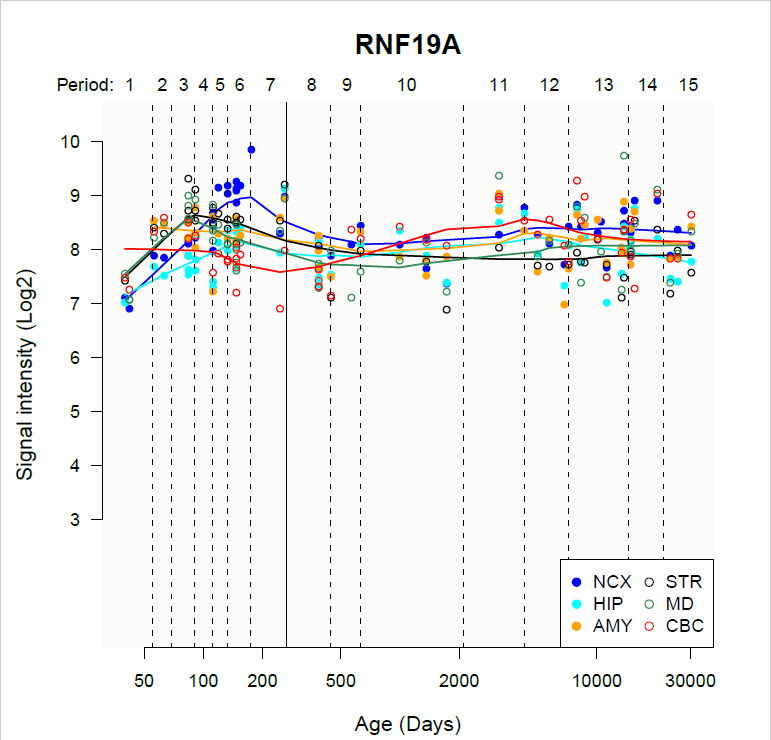
**

**Supplementary Figure 5: Network diagram generated by Ingenuity Pathway Analysis (IPA) (European ancestry).** Shaded genes indicate those that were identified as focus or input genes for the IPA network analysis in either the single variant or gene-based meta-analyses of cohort-specific GWAS of processing speed after adjustment for age and gender, or in the secondary analyses that also included education (p < 1 x 10^-4^). Direct interactions between genes are depicted by solid lines, and indirect interactions are shown by dotted lines.


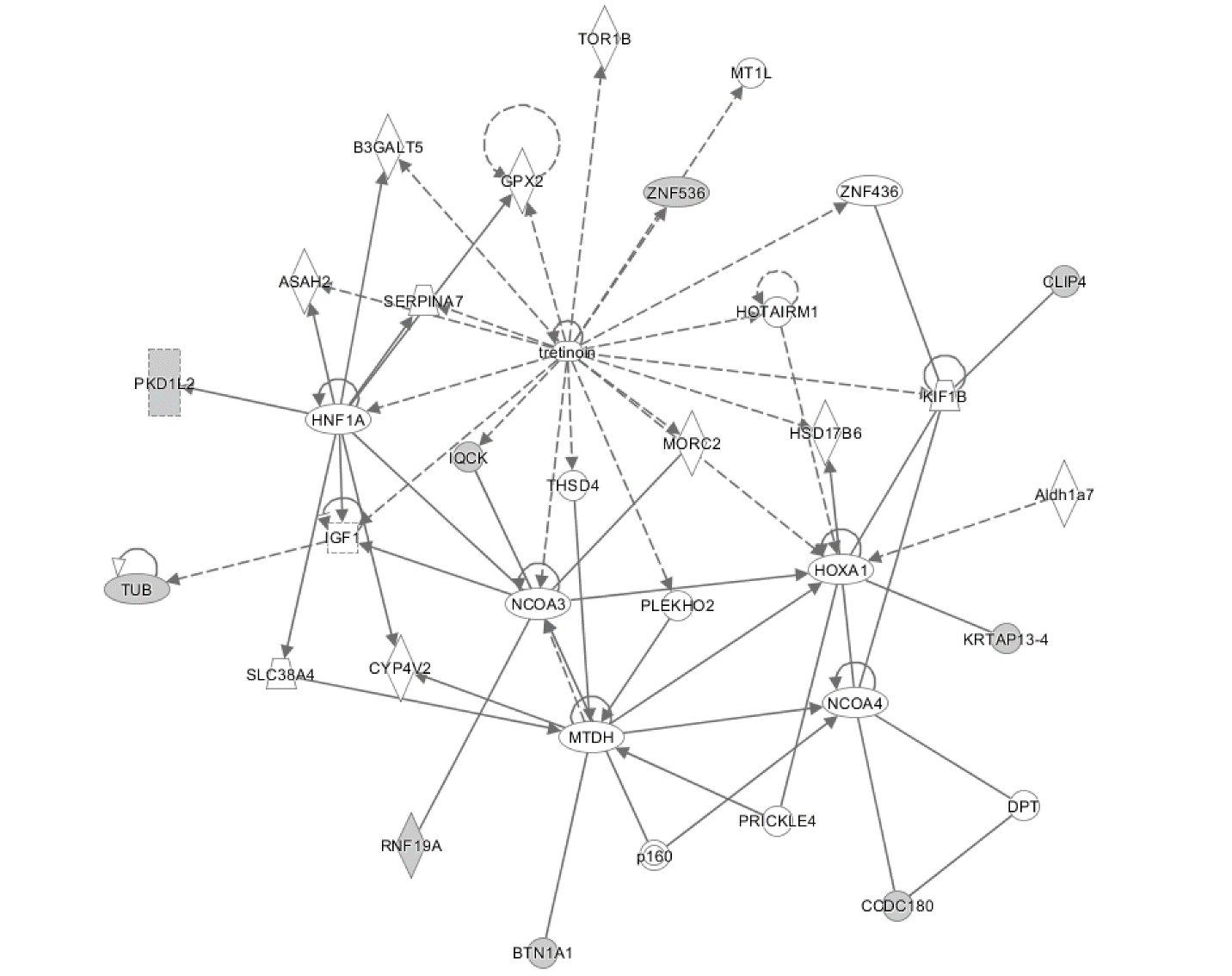


**Supplementary Figure 6: Network diagram generated by Ingenuity Pathway Analysis (IPA) (African-American).** Shaded genes indicate those that were identified as focus or input genes for the IPA network analysis in either the single variant or gene-based meta-analyses of cohort-specific GWAS of processing speed after adjustment for age and gender, or in the secondary analyses that also included education (p < 1 x 10^-4^). Direct interactions between genes are depicted by solid lines, and indirect interactions are shown by dotted lines.
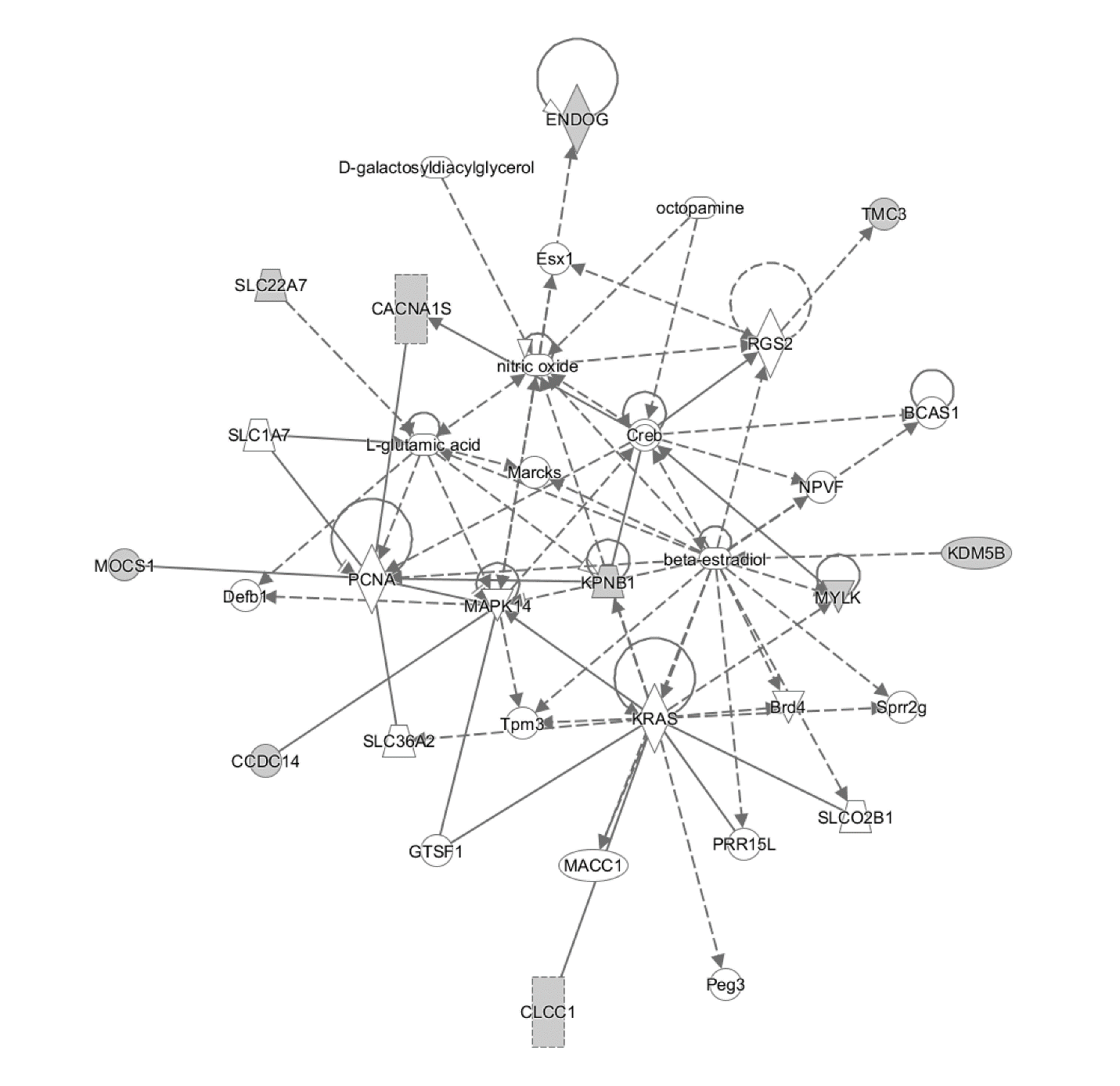


**4. Acknowledgements**

**Age, Gene/Environment Susceptibility-Reykjavik Study (AGES-Reykjavik)**: This study has been funded by NIH contract N01-AG-12100 with contributions from NEI, NIDCD and NHLBI, the NIA Intramural Research Program, Hjartavernd (the Icelandic Heart Association), and the Althingi (the Icelandic Parliament). The study was approved by the Icelandic National Bioethics Committee (VSN: 00-063). The researchers are indebted to the participants for their willingness to participate in the study.

**Atherosclerosis Risk in Communities (ARIC) Study:** The Atherosclerosis Risk in Communities study has been funded in whole or in part with Federal funds from the National Heart, Lung, and Blood Institute, National Institutes of Health, Department of Health and Human Services (contract numbers HHSN268201700001I, HHSN268201700002I, HHSN268201700003I, HHSN268201700004I and HHSN268201700005I). The authors thank the staff and participants of the ARIC study for their important contributions. Funding support for “Building on GWAS for NHLBI-diseases: the U.S. CHARGE consortium” was provided by the NIH through the American Recovery and Reinvestment Act of 2009 (ARRA) (5RC2HL102419).

**Austrian Stroke Prevention Study (ASPS):** The ASPS is funded by the Austrian Science Fund (FWF) grant numbers P20545-B05 to H.S., P13180 and P1904 to R.S., the Austrian National Bank Anniversary Fund grant number P15435 to H.S., and the Austrian Ministry of Science under the aegis of the EU Joint Programme-Neurodegenerative Disease Research (JPND)-www.jpnd.eu through BRIDGET to H.S. The Medical University of Graz supports the Ph.D. program “Molecular Medicine”. The authors thank the staff and the participants for their valuable contributions. We thank Birgit Reinhart for her long-term administrative commitment, Elfi Hofer for the technical assistance at creating the DNA bank, Ing. Johann Semmler and Anita Harb for DNA sequencing and DNA analyses by TaqMan assays and Irmgard Poelzl for supervising the quality management processes after ISO9001 at the biobanking and DNA analyses.

**Cardiovascular Health Study (CHS):** This research was supported by NHLBI contracts HHSN268201200036C, HHSN268200800007C, HHSN268201800001C, N01HC55222, N01HC85079, N01HC85080, N01HC85081, N01HC85082, N01HC85083, N01HC85086, 75N92021D00006; and NHLBI grants U01HL080295, R01HL087652, R01HL105756, R01HL103612, R01HL120393, and R01HL130114 with additional contribution from the National Institute of Neurological Disorders and Stroke (NINDS). Additional support was provided through R01AG023629, R01AG033193, R01AG15928, R01AG20098, from the National Institute on Aging (NIA), and U54HG003273 from the National Human Genome Research Institute (NHGRI). Funding support for “Building on GWAS for NHLBI-diseases: the U.S. CHARGE Consortium” wan provided by NIH through the American Recovery and Reinvestment Act of 2009 (ARRA) (5RC2HL102419). The provision of genotyping data was supported in part by the National Center for Advancing Translational Sciences, CTSI grant UL1TR0001881, and the National Institute of Diabetes and Digestive and Kidney Diseases Diabetes Research Center (DRC) grant DK063491 to the Southern California Diabetes Endocrinology Research Center. A full list of principal CHS investigators and institutions can be found at http://www.chs-nhlbi.org/pi. The content is solely the responsibility of the authors and does not necessarily represent the official views of the National Institutes of Health.

**Coronary Artery Risk Development in Young Adults (CARDIA):** This study is conducted and supported by the National Heart, Lung, and Blood Institute (NHLBI) in collaboration with the University of Alabama at Birmingham (HHSN268201800005I & HHSN268201800007I), Northwestern University (HHSN268201800003I), University of Minnesota (HHSN268201800006I), and Kaiser Foundation Research Institute (HHSN268201800004I). CARDIA was also partially supported by the Intramural Research Program of the National Institute on Aging (NIA) and an intra-agency agreement between NIA and NHLBI (AG0005). Exome chip genotyping and data analyses were funded in part by grants U01-HG004729 and R01-HL093029 to M.F., R01-NS087541 to M.F. and E.B., and RO1-HL122658 to K.Y. from the National Institutes of Health. This manuscript has been reviewed by CARDIA for scientific content.

**CROATIA– Korcula study (Korcula):** The CROATIA-Korcula study on the Croatian island of Korcula was supported through grants from the Medical Research Council UK and the Ministry of Science, Education and Sport in the Republic of Croatia (number 108-1080315-0302), the Croatian National Centre of Research Excellence in Personalized Healthcare grant (number KK.01.1.1.01.0010), and the Centre of Competence in Molecular Diagnostics (KK.01.2.2.03.0006). Exome array genotyping was performed at the Clinical Research Facility, University of Edinburgh, Edinburgh, UK. We would like to acknowledge the invaluable contributions of the recruitment team in Korcula, the administrative teams in Croatia and Edinburgh and the people of Korcula.

**Framingham Heart Study (FHS)**: The Framingham Heart Study is conducted and supported by the National Heart, Lung, and Blood Institute (NHLBI) in collaboration with Boston University (Contract No. N01-HC-25195, HHSN268201500001I and 75N92019D00031). Funding for SHARe Affymetrix genotyping was provided by NHLBI Contract N02-HL-64278. This work was also supported by grant 5R01AG054076 from the National Institute on Aging to S.S., grant 5R01NS017950 from the National Institute of Neurological Disorders and Stroke to S.S., and grant 5R01NS087541 from the National Institute of Neurological Disorders and Stroke to M.F. and E.B. The computational work reported in this paper was performed on the Shared Computing Cluster which is administered by Boston University’s Research Computing Services. We thank all the FHS study participants and staff to make this research possible.

**Genetic Study of Atherosclerosis Risk (GeneSTAR)**: GeneSTAR was supported by grants from the National Institutes of Health National Institute of Neurological Disorders and Stroke (R01NS062059), the National Institutes of Health National Heart, Lung, and Blood Institute (U01 HL72518, HL087698), the National Institutes of Health/National Center for Research Resources (M01-RR000052) to the Johns Hopkins General Clinical Research Center, and National Institutes of Health/National Center for Research Resources and the National Center for Advancing Translational Sciences (UL1 RR 025005) to the Johns Hopkins Institute for Clinical and Translational Research. This work was also supported by grant RO1NS062059 from the National Institute of Neurological Disorders and Stroke to P.N. We would like to thank the participants and families of GeneSTAR and our dedicated staff for all their sacrifices.

**Generation Scotland: Scottish Family Health Study (GS:SFHS):** Generation Scotland received core funding from the Chief Scientist Office of the Scottish Executive Health Directorate CZD/16/6. Exome array genotyping for GS:SFHS was funded by the Medical Research Council UK and performed at the Clinical Research Facility, University of Edinburgh, UK. Genotyping of the GS:SFHS samples was carried out by the Genetics Core Laboratory at the Edinburgh Clinical Research Facility, University of Edinburgh, Scotland and was funded by the Medical Research Council UK and the Wellcome Trust (Wellcome Trust Strategic Award “Stratifying Resilience and Depression Longitudinally (STRADL) Reference 104036/Z/14/Z).” We would like to acknowledge the invaluable contributions of the families who took part in the Generation Scotland: Scottish Family Health Study, the general practitioners and Scottish School of Primary Care for their help in recruiting them, and the whole Generation Scotland team, which includes academic researchers, IT staff, laboratory technicians, statisticians and research managers. C.H. is supported by an MRC University Unit Programme Grant MC_UU_00007/10 (QTL in Health and Disease).

**Genetic Epidemiology Network of Arteriopathy (GENOA):** Support for GENOA was provided by the National Heart, Lung and Blood Institute (HL119443, HL054464, HL054457, HL054481, and HL087660) and the National Institute of Neurological Disorders and Stroke (NS041558) of the National Institutes of Health. Genotyping was performed at the Mayo Clinic and was made possible by the University of Texas Health Science Center. We would also like to thank the families that participated in the GENOA study.

**Lothian Birth Cohort 1921 (LBC1921) and Lothian Birth Cohort 1936 (LBC1936)**: We thank the cohort participants and team members who contributed to these studies. Phenotype collection in the Lothian Birth Cohort 1921 was supported by the UK Biotechnology and Biological Sciences Research Council (BBSRC), The Royal Society and The Chief Scientist Office of the Scottish Government. Phenotype collection in the Lothian Birth Cohort 1936 was supported by Research Into Ageing (continues as part of Age UK The Disconnected Mind project). Genotyping of the cohorts was funded by the BBSRC. The work was undertaken by The University of Edinburgh Centre for Cognitive Ageing and Cognitive Epidemiology, part of the cross council Lifelong Health and Wellbeing Initiative (MR/K026992/1). Funding from the BBSRC and Medical Research Council (MRC) is gratefully acknowledged.

**Rotterdam Study (RS):** The Rotterdam Study is funded by Erasmus Medical Center and Erasmus University, Rotterdam, Netherlands Organization for the Health Research and Development (ZonMw), the Research Institute for Diseases in the Elderly (RIDE), the Ministry of Education, Culture and Science, the Ministry for Health, Welfare and Sports, the European Commission (DG XII), and the Municipality of Rotterdam. The generation and management of GWAS genotype data for the Rotterdam Study is supported by the Netherlands Organisation of Scientific Research NWO Investments (nr.175.010.2005.011, 911-03-012). This study is funded by the Research Institute for Diseases in the Elderly (014-93-015; RIDE2); the Netherlands Genomics Initiative (NGI)/Netherlands Organisation for Scientific Research (NWO) Netherlands Consortium for Healthy Aging (NCHA) project nr. 050-060-810; the European Union’s Horizon 2020 research and innovation programme (project: CoSTREAM), grant number 667375; and the European Research Council (ERC) under the European Union’s Horizon 2020 research and innovation programme (project: ORACLE), grant number 678543. We thank Pascal Arp, Mila Jhamai, Marijn Verkerk, Lizbeth Herrera, Marjolein Peters and Carolina Medina-Gomez for their help in creating the GWAS database, and Karol Estrada, Yurii Aulchenko, and Carolina Medina-Gomez for their support in creation and analysis of imputed data. The authors are grateful to the study participants, the staff from the Rotterdam Study and the participating general practitioners and pharmacists.
